# Health-related quality of life outcomes in randomized controlled trials in metastatic hormone-sensitive prostate cancer: a systematic review

**DOI:** 10.1016/j.eclinm.2024.102914

**Published:** 2024-11-13

**Authors:** Susanne Osanto, Anne-Laurien van de Vliert-Bout, Cristina Alvarez Gomez de Segura, Fabio Efficace, Francesco Sparano, Peter-Paul Willemse, Jan Schoones, Adam Cohen, Sahar Barjesteh van Waalwijk van Doorn-Khosrovani

**Affiliations:** a Department of Clinical Oncology, Leiden University Medical Center (LUMC), the Netherlands; b Department of Urology, Leiden University Medical Center; Current Affiliation ELEOS Mental Health Care Institution, Gouda, the Netherlands; c Italian Group for Adult Hematologic Diseases (GIMEMA) Data Center and Health Outcomes Research Unit, Rome, Italy; d Department of Urology, Utrecht Medical Center, Utrecht, the Netherlands; e Directorate Research Policy, Leiden University Medical Center (LUMC), the Netherlands; f Center for Human Drug Research (CHDR), Leiden, the Netherlands; g CZ Health Insurance, Tilburg, the Netherlands

**Keywords:** Metastatic hormone-sensitive prostate cancer (mHSPC), Health-related quality of life (HRQoL), Clinical trial, Systematic review, Clinical decision-making

## Abstract

**Background:**

Since 2015 multiple combination treatments became available for metastatic hormone-sensitive prostate cancer (mHSPC) without effectiveness cross-comparison. Health-related quality of life (HRQoL) could aid in decision-making.

**Methods:**

We systematically reviewed HRQoL publications (January 2015–September 2024) of phase III randomized controlled trials (RCTs) in mHSPC using PRISMA guidelines, cross-compared HRQoL results and assessed usefulness to support decision-making (PROSPERO: CRD42023470698). International Society for Quality-of-Life Research (ISOQOL) recommended standards were used to assess quality of Patient-reported Outcomes reporting.

**Findings:**

We identified nine HRQoL publications from eight RCTs investigating an estradiol patch, or either radiotherapy, docetaxel, androgen-receptor-pathway-inhibitor (ARPI) abiraterone, apalutamide or enzalutamide added to androgen deprivation therapy (ADT) versus ADT ± placebo in ≥8000 patients. Only three studies were considered to have low overall risk of bias (RoB2). Eight HRQoL measures (1–4 per study) were used; 3/5 RCTs investigating an ARPI measured HRQoL using Brief Pain Inventory (BPI-SF), and Functional Assessment of Cancer Therapy-Prostate (FACT-P). Overall, the quality of PRO reporting was high, but PRO-hypothesis was provided by only 25% and reasons for missing data explained in only 50% of RCTs.

**Interpretation:**

Conceptual and methodological HRQoL heterogeneity, along with risk of biases, hampers cross-comparison and failed to robustly support decision-making underscoring the importance of harmonizing methodological approaches.

**Funding:**

None.

**Research in context**

**Evidence before this study**

Since 2015, phase III randomized controlled trials (RCTs) in metastatic hormone-sensitive prostate cancer (mHSPC) have been published which for the first time demonstrated improved survival of combination therapies compared to androgen deprivation therapy (ADT) alone, which had been the standard of care for decades.

We conducted a systematic review of HRQoL reports of mHSPC phase III RCTs, retrieved from PubMed, Embase (OVID), Web of Science, Cochrane Library, and Google Scholar, and published between January 2015 and September 1st, 2024.

**Added value of this study**

Unlike previous reviews of contemporary phase III RCTs in mHSPC that focused on presence of high-volume metastatic disease and traditional oncological clinical outcome measures our systematic review focusses on HRQoL reporting. HRQoL publications revealed significant methodological heterogeneity in HRQoL studies, variations in HRQoL measures, individual HRQoL endpoints, data collection time points and statistical methods used for PRO analysis. In addition, the study identifies risks of bias, such as lack of blinding, absence of a priori hypotheses, lack of statistical power and issues related to handling of missing data.

**Implications of all the available evidence**

The study demonstrated conceptual and methodological differences across trials and highlights the urgent need for standardisation and harmonization of HRQoL methods in prostate cancer research. HRQoL standardisation will enhance reliability of results, facilitate cross-comparisons, and drive utilization of PRO outcomes in healthcare systems supporting better-informed decision-making in patient care.

## Introduction

In oncology, clinical trial endpoints focus on measurable disease- and survival-related endpoints as benchmark of benefit, thus forming the core of regulatory decision-making. Health-related quality of life (HRQoL) data obtained by validated patient reported outcome (PRO) measures provide the unique patient's perspective on the burden of cancer treatment. HRQoL is a dynamic endpoint of treatment benefit rather than overall survival (OS) endpoint and could potentially support the benefit/risk ratio of intervention treatment evaluation. HRQoL balances between symptoms related to the disease, disease control by effective treatment and treatment-associated side effects. However, to generate high-quality PRO data that can successfully support clinical decision-makings, several aspects regarding the HRQoL protocol design and outcome reporting are to be thoughtfully considered. Key questions are whether the investigational treatment arm improves QoL or delays deterioration and whether QoL is better, worse than, or non-inferior to QoL associated with the standard of care (SOC).

The American Society of Clinical Oncology (ASCO) and European Society of Medical Oncology (ESMO) recognize QoL as one of the parameters for the evaluation of clinical value of anticancer treatments and recommend incorporation of quality-of-life measurements as part of the endpoints of clinical trials.^1^, ^2^, ^3^, ^4^

The US Food and Drug Administration (FDA) and the European Medicines Agency (EMA) are both supportive of the use of patient reported outcomes (PROs) in anticancer drug development. In 2009 the United States Food and Drug Administration (FDA) published a guidance to streamline the use of PROs in clinical trials,^5^ which was further extended and updated in 2018 and 2021.^6^^,^^7^ In 2016 EMA published a guidance to inclusion of PRO in clinical research and evaluation of anticancer medicinal products.^8^ ESMO also provided guidance for methodological and reporting standards for quality-of-life data eligible for European Society for Medical Oncology-Magnitude of Clinical Benefit Scale (ESMO-MCBS) credit.^9^

For decades, androgen deprivation therapy (ADT) was the mainstay in metastatic hormone-sensitive prostate cancer (mHSPC). Most trials were performed upon progression from metastatic hormone-sensitive to metastatic castration-resistant prostate cancer (mCRPC) and these investigated combinations of one drug added to continued ADT versus continued ADT. This led in 2004 to FDA drug approval of docetaxel for treatment of mCPRC patients (Supplementary Table S1) with more FDA drug approvals for mCRPC in the years to follow.

It took till 2015 for a breakthrough to occur in the early metastatic hormone-sensitive space when the clinical success of the CHAARTED trial investigating the combination of docetaxel plus ADT versus ADT alone as first-line treatment in metastatic hormone sensitive prostate cancer was presented at international congresses. Subsequently, other trials followed and the first FDA drug approval for first-line mHSPC treatment was in 2018 (abiraterone plus prednisone in combination with ADT) (Supplementary Table S1).

Thus, a number of phase III RCTs that have been published since 2015, demonstrated an OS benefit of investigational combinations plus ADT compared to ADT with or without placebo, showing similar hazard ratios of death.^10^^,eFig.S3^ This changed the treatment landscape of mHSPC, and multiple combinations of ADT plus another anticancer drug became available for the treatment of mHSPC patients. The lack of head-to-head comparisons between investigational treatments and the greatly varying drug costs of the novel combination treatments posed challenges to clinicians in clinical decision-making. Hence, the availability of HRQoL in this area could provide key information to make more informed treatment decisions amongst the multiple treatment options available. However, several methodological aspects are to be well addressed to ensure that HRQoL data can provide meaningful insights in prostate cancer RCTs.^11^

We therefore performed a systematic literature review of HRQoL publications of III RCTs conducted in patients with mHSPC to cross-compare their results and assess whether QoL results provide guidance to clinicians and patients in their choices between novel combination treatments.

We evaluated the quality of PRO reporting in phase III RCTs that investigate new treatments versus ADT in patients with mHSPC, and compared PROs, including statistically and clinically meaningful differences, between treatment arms across phase III RCTs.

## Methods

### Literature search strategy and data extraction

A systematic literature search was conducted by a trained medical librarian (JWS) together with two reviewers (AvdV-B, SO). The databases searched were PubMed, Embase (OVID-version), Web of Science, Cochrane Library, and Google Scholar (Supplementary Table S2). The query consisted of the combination of the following four concepts: 1) Metastatic Hormone Sensitive Prostate Cancer, 2) Androgen Deprivation Therapy, 3) Quality of Life or patient reported outcome (PRO) Measures and 4) Phase III randomized controlled trials (RCT). Eligibility criteria were Health-related quality of life (HRQoL, QoL) publications of phase III RCT's investigating new systemic treatment in comparison to androgen deprivation therapy (ADT) standard of care in mHSPC. The selection period of publications was from January 1, 2015 to September 1, 2024.

**Fig. 1:**
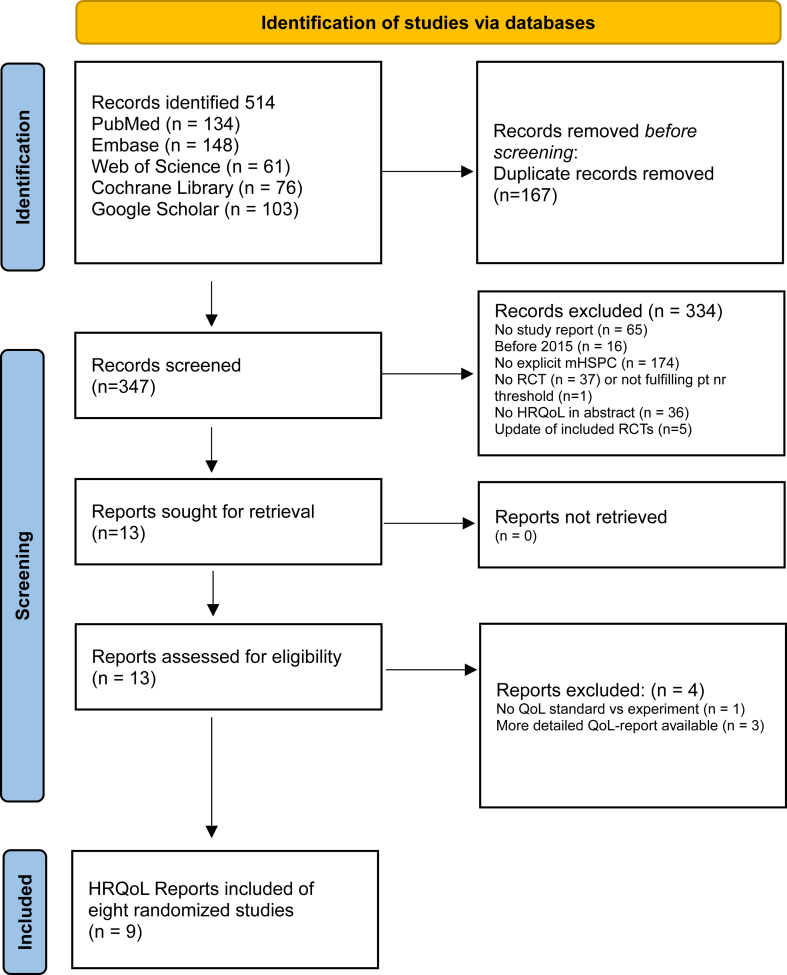
**Prisma flowchart of study selection**.

Relevant keyword variations in the controlled vocabularies of the various databases, and the free text word variations of these concepts, were used. We excluded publications in which the HRQoL analysis was based on 50 patients or less (as a threshold). Meeting abstracts were excluded, and results were limited to the English language. The results were limited to articles published from 2015 onwards. The final search was performed September 01, 2024. A standardised data extraction form was developed and before its use piloted by two review authors (AvdV-B and CA) used to extract data from eligible studies. No automation tools were used to collect data, no software was used to extract data from figures. Data extraction followed three subsequent steps of revisions following the Preferred Reporting Items for Systematic Review and Meta-Analyses (PRISMA) statement^12^ (Fig. 1). Two reviewers (AvdV-B, SO) independently conducted eligibility screening of publications to identify the final included PRO publications of RCTs. In the first step, titles and abstracts of search results based on inclusion and exclusion criteria were screened, references retrieved in full text and evaluated. Extracted data were compared, with any discrepancies being resolved through discussion. Disagreements were resolved by consensus (AvdV-B, SO). When information regarding any of the above was unclear, authors of the reports were contacted to provide further details.

### Quality assessment and data analysis

The study protocol was registered in PROSPERO (the International Prospective Register of Systematic Reviews, CRD42023470698; Supplementary Appendix 1).

We selected HRQoL publications from phase III RCTs in mHSPC and assessed the quality of PRO reporting using International Society for Quality-of-Life Research (ISOQOL) Consortium recommendations.^13^, ^14^, ^15^ ISOQOL was not only evaluated based on the HRQoL publication but also on the clinical protocol published as Supplementary material in the clinical outcome publication or otherwise made publicly available.

We focussed on presence of prespecified objectives (hypothesis, primary, secondary or exploratory endpoints), analytical methods, uniformity in PRO investigations and reporting, clarity of PRO data provided (e.g. PRO outcome bias due to the open label study design, compliance after baseline evaluation) and whether outcomes were reported at the same time with PFS and/or OS results.

The quality of methodology, risk of bias assessments of retrieved articles, and comparisons made across studies. For cross-study comparisons, we studied baseline QoL of the patient populations across RCTs to assess QoL homogeneity of the different mHSPC trial populations.

### Assessment of risk of bias

The risk of bias of each study was assessed by (two) review authors working independently. Any disagreements were resolved by consensus or by consulting a third review author if necessary. The quality of included publications was assessed using the risk of bias tool of the Revised Cochrane risk-of-bias tool for randomized trials (Risk of Bias, RoB2).^16^ Following guidance given for RoB 2.0, we derived an overall summary ‘Risk of Bias' judgement (low; some concerns; high) for each specific outcome, whereby the overall RoB for each study was determined by the highest RoB level in any of the domains that were assessed.

### ISOQOL

The quality of PRO reporting was reviewed by using the ISOQOL recommended standards.^13^^,^^17^ These standards consist of 18 items that are to be addressed for all RCTs, regardless of PRO being primary or secondary endpoint. Using these recommended standards, for each RCT we assessed whether, for example, a PRO hypothesis was stated, missing PRO data reported, or statistical approaches for dealing with missing data reported.

### Statistics

Descriptive data are reported as frequencies and percentages. Text and table format are used to provide a qualitative overview of the findings.

### Ethics statement

This study used published data from existing studies. The requirement for ethical approval and informed consent were waived for this study.

### Role of the funding source

There was no funding source for this study. All authors had access to the data and all authors were responsible for making the decision to submit this manuscript.

## Results

### Study characteristics

A total of nine peer reviewed HRQoL publications from eight RCTs, including one randomized controlled trial using a multi-arm, multi-stage platform design, appearing between January 2015 and September 2024^18^, ^19^, ^20^, ^21^, ^22^, ^23^, ^24^, ^25^, ^26^ from phase III RCTs in mHSPC (PATCH; https://clinicaltrials.gov/ct2/show/NCT00303784)^27^, ^28^, ^29^, ^30^, ^31^, ^32^, ^33^, ^34^, ^35^, ^36^ were identified according to our predefined selection criteria (Table 1). Two publications came from STAMPEDE, a multi-arm study

**Table 1:** Summary of HRQoL studies published between January 2015 and September 2024 of phase III RCTs in mHSPC.

RCT	PATCH	LATTITUDE	CHAARTED	TITAN	ARCHES	HORRAD	STAMPEDE		ENZAMET
**Clinical outcome publication of phase III RCT**									
Publication year	NA∗	2017	2015	2019	2019	2019	2018	2018	2019
1st Author	https://clinicaltrials.gov/ct2/show/NCT00303784	Fizazi et al.^27^	Sweeney et al.^28^	Chi et al.^29^	Armstrong et al.^30^	Boevé et al.^31^	James et al.,^32^^,^^33^ Sydes et al.^34^	Parker et al.^35^	Davis et al.^36^
Sample size	1694	1199	790	1052	1150	432	566 (2011–2013)	2061	1125
Randomization	2:1	1:1	1:1	1:1	1:1	1:1	1:1	1:1	1:1
Experimental Arm	tOestradiol	ADT + AAP	ADT + DOC	ADT + APA	ADT + ENZ	ADT + EBRT	ADT + DOC; ADT + AAP	ADT + EBRT	ADT + ENZ
Control Arm	ADT	ADT + PBO	ADT	ADT + PBO	ADT + PBO	ADT	ADT	ADT	ADT
Primary endpoints	CVD morbidity, mortality; QoL outcomes	OS, rPFS	OS + time to clinical progression	OS, rPFS	rPFS	OS	OS, PFS	OS	OS
Outcome	Not Reported	OS benefit	OS benefit	OS benefit	rPFS benefit	No OS benefit	OS benefit	OS benefit	OS benefit
**HRQoL publication of the phase III RCT**									
Publication year	2017	2018	2018	2019	2020	2021	2022	2022	2022
1st Author	Gilbert et al.^18^	Chi et al.^19^	Morgans et al.^20^	Agarwal et al.^21^	Stenzl et al.^22^	Boevé et al.^23^	Rush et al.^24^	Parker et al.^25^	Stockler et al.^26^
Time period	August 14, 2007–October 5, 2015	February 12, 2013–December 11, 2014	July 2006–December 2012	December 2015–July 2017	March 2016–January 2018	November 2004–September 2014	November 2011–March 2013	January 2013–September 2016	March 2014–March 2017
Sample size	727	1149	706	1016	1085	423	515	2061	1042
Endpoints of RCT	2nd	2nd	2nd	Exploratory	2nd	2nd	2nd	2nd	2nd
QoL tools used, nr	2	4	4	4	4	2	2	1	3
	EORTC-QLQ-C30 EORTC-QLQ-PR25	FACT-P EQ-5D-5L BPI-SF BFI-SF	FACT-P FACT-Taxane BPI-SF FACIT-F	FACT-P EQ-5D-5L BPI-SF BFI-SF	FACT-P EQ-5D-5L BPI-SF EORTC-QLQ-PR25	EORTC-QLQ-C30 EORTC-QLQ-PR25	EORTC-QLQ-C30 EORTC-QLQ-PR25	EORTC-QLQ-C30	EORTC-QLQ-C30 EORTC-QLQ-PR25∗ EQ-5D-5L
Time points next to baseline	Longitudinal till 2 year. 6 mo reported in 2017	Longitudinal until EOT. Only EQ-5D-5 L till 1 yr after the EOT.	Longitudinal: 3, 6, 9 and 12 months	Longitudinal until progression, pain ao after progression.	Longitudinal: until disease progression	Longitudinal: 3, 6, 12, and 24 months	Longitudinal, annually after 5 yr	Longitudinal till 2 year. Global QoL % and QLQ-30 Summary. Cross-sectional 12, 24, 60 and 104 wks.	Longitudinal weeks 4 and 12, and then every 12 weeks until clinical progression
Compliance at baseline and during treatment.	E: 90% at BL and 88% at 6 mo; C: 93% at BL and 91 at 6 mo	E and C: 96% at BL and 90% or higher for all PRO measurement tools	E: 92% at BL and 77% at 12 mo C: 88% at BL and 76% at 12 mo	75–95% before cycle 13; 75–90% after cycle 13 for PRO tools	E: 94–96% at BL and 87% at wk 73; C: 95–96% at BL and 88% at wk 73	E and C: 98% at BL, declining to 58% at 2 yr.	DOC and AAP: 98% at baseline, and 67% and 75% over 2 yr	Not provided	E and C: 100% at BL and 86% at week 156
QoL in exp. Arm versus ADT control arm	Better 6-month QoL outcomes	Better overall PROs, pain progression, PCa symptoms, fatigue, etc.	Worse at 3 mo, better at 12 mo	HRQoL maintained	High-functioning HRQoL maintained	More temp. urinary + bowel symptoms.	Global QoL AAP higher than DOC	Only differences in mean estimates provided at time points	Worse fatigue, cognitive, physical function, not Global QoL

**Abbreviations:** FU, follow up; PBO, placebo; OS, overall survival; rPFS, radiological progression free survival; tOestradiol or tE2, transdermal oestradiol patch; ADT, androgen deprivation therapy; ADT, (LHRH receptor agonist or an LHRH receptor antagonist or orchiectomy); ADT+AAP, ADT plus abiraterone and predniso(lo)n; ADT+DOC, ADT plus docetaxel chemotherapy; ADT+APA, ADT plus apalutamide; ADT+ENZ, ADT plus enzalutamide; ADT+EBRT, ADT plus external beam radiotherapy; CVD, Cardiovascular disease; NA, not available; NR, not reached; mHSPC, metastatic hormone-sensitive prostate cancer; HR, hazard ratio for risk of death and 95% CI; CI, confidence interval; # EORTC-PR25 or QLM-PR25l (named in ENZAMET); CVD, cardiovascular disease; BL, at baseline; EOT, end of treatment. a.o., amongst others.

PATCH: Only 40% had metastatic disease (mHSPC), while 60% had locally advanced disease (nmHSPC). Phase 2 and 3 part of the study were combined. tE2 was delivered via three or four transcutaneous patches containing oestradiol 100 g/24 h, versus LHRHa for ADT. LHRHa = luteinizing hormone-releasing hormone (LHRH) antagonist, was administered as per local practice.

∗) The progression-free and overall survival of the 1694 patients in the PATCH study have not been reported yet. The long-term follow-up showed no difference in cardiovascular mortality or morbidity between transdermal oestradiol and ADT (Langley RE, Gilbert DC, Duong T et al. Lancet 2021; 397(10274):581-591. doi: 10.1016).

LATITUDE: High-risk was defined as meeting at least two of three criteria: (i) Gleason score ≥8, (ii) presence of ≥3 lesions on bone scan, or (iii) presence of measurable visceral lesions. ADT consisted of LHRH or surgical castration. ADT + Abiraterone 1000 mg/day and Prednisone mg/day (5 mg daily) (the abiraterone group); ##After the results of the first interim analysis, the LATITUDE study was unblinded and patients in the placebo group were allowed to cross over to receive abiraterone acetate and prednisone plus ADT treatment in accordance with a protocol amendment (February 2017) in an open-label extension of the study.

CHAARTED (“CHAARTED, Chemo Hormonal Therapy versus Androgen Ablation Randomized Trial for Extensive Disease in Prostate Cancer”): High-volume was defined as: Visceral Metastases AND/OR ≥4 Bone Lesions (with ≥1 beyond the vertebral bodies and pelvis). DOC + ADT (docetaxel 75 mg/m2, q3 wks for 6 cycles). Daily prednisolone not required. Steroids only given as premedication. Use of GCSF was permitted. versus ADT (LHRH receptor agonist or an LHRH receptor antagonist or orchiectomy; antiandrogens were given at the investigators' decision). ADT (LHRH receptor agonist or an LHRH receptor antagonist or orchiectomy; antiandrogens were given at the investigators' decision). Docetaxel (DOC) is given as 75 mg/m2 IV 3-weekly for six cycles (Daily prednisolone not required in CHAARTED, Steroids were only given as premedication (8 mg of oral dexamethasone at 12 h, 3 h, and 1 h before docetaxel infusion). Use of GCSF was permitted; CHAARTED: prior adjuvant ADT was allowed if the duration of therapy was 24 months or less and progression had occurred more than 12 months after completion of therapy.

ARCHES: Enzalutamide is a 2nd generation androgen receptor pathway inhibitor (ARPI) and is given continuous oral 160 mg daily. Prior docetaxel of ADT were allowed.

TITAN: Apalutamide is a 2nd generation/androgen receptor pathway inhibitor (ARPI) and is given continuous oral 240 mg daily; After unblinding of the study, patients on ADT (with placebo) were allowed to cross over to APA.

HORRAD mHSPC with the primary tumour in situ. ∗Addition of RT to the primary did not improve OS (HR 0.90; 95% CI, 0.70–1.14), but an unplanned subset analysis demonstrated that patients with low volume disease had a non-significant longer OS (HR 0.68; 95% CI, 0.42–1.10).

STAMPEDE (STAMPEDE, Systemic Therapy in Advancing or Metastatic Prostate Cancer: Evaluation of Drug Efficacy): Newly diagnosed mHSPC N+, or high risk locally advanced N0M0, ≥2 of following T3 or T4, GS ≥ 8, PSA ≥40 ng/ml, or recurrent disease after local therapy with high-risk features of metastasis.

Docetaxel (DOC) is given as 75 mg/m2 IV 3-weekly for six cycles, steroids (prednisolone/prednisone 10 mg daily) is given during the docetaxel treatment period.

STAMPEDE: Abiraterone acetate (AA), is an androgen biosynthesis inhibitor, and is given continuous oral in a dose of 1000 mg daily with prednisolone/prednisone 5 mg daily.

In ENZAMET, patients were treated with docetaxel given every 3 weeks for a maximum of six cycles, but up to two cycles of docetaxel were permitted before randomization. Testosterone suppression initiated up to 12 wks before randomization; administration of docetaxel was allowed.

For additional detailed information about each RCT please see Supplementary Appendix 2 Clinical trial information.

Three trials (LATITUDE, TITAN and ARCHES (Table 1) were pharma-initiated, blinded, using matching placebos added to ADT in the control arm, executed globally and recruited in just 2 years. Five were investigator-initiated, open-label, originating in 4 different countries (PATCH, multi-arm, multistage platform design study STAMPEDE, CHAARTED, HORRAD and ENZAMET) (Table 1), 3 of which started before 2010 and recruited patients over an extended period.

HRQoL was a secondary endpoint (n = 7) or exploratory endpoint (n = 1) of the trials (Table 1) and outcomes were published with a delay of 4 months to 4 years after the primary clinical outcome publications. HRQoL was reported using eight validated questionnaires, ranging from 1 to 4 instruments per study, in different combinations. QoL was collected longitudinally at different timepoints, for different time periods (Table 1, Table 2, Table 3, Table 4; Supplementary Table S3).

**Table 2:** QoL outcomes of 8 included phase 3 RCTs in mHSPC.

	Measured	Arm Type	FACT-P^c^	FACT-Taxane	FACIT-Fatigue	BPI	BPI	BPI	EQ-5D-5L VAS	BFI^d^	EORTC QLQ-C30	EORTC-PR25
			Total			worst	severity	interference		worst	Global	
**PATCH**												
BL	Mean	C	–	–	–	–	–	–	–	–	**75.1**	Less hot flushes **(P < 0.0001)**^b^, more gynecomastia **(P < 0.001)**^b^, not at all interested in sex **(P < 0.001)**^b^ in tE2 arm. No further significant differences found.
6 mo			–	–	–	–	–	–	–	–	70.1	
BL	Mean	E	–	–	–	–	–	–	–	–	**78.0**	
6 mo											75.2	
6 mo	Estimated mean difference P value^a^		–	–	–	–	–	–	–	–	+4.2 (95% CI 1.2–7.1; **P = 0.006**^b^) in favour of tE2.	
**LATITUDE**												
BL	Mean (sd)	C		–	–	2.2 (2.4)	–	1.5 (2.0)	73.9 ± 17.6	NR-NR 25th percentile: 6.5 (5.6–9.2)	–	–
Results	MTDD Median months (95% BI)		12.9 (9.0–16.6)	–	–	NR-NR 25th percentile: 11.07 (9.23–18.43)	–	NR-NR 25th percentile: 6.5 (4.6–9.2)	Results in figure only		–	–
BL	Mean (sd)	E		–	–	2.2 (2.5)	–	1.5 (2.0)	74.2 ± 16.8	**NR-NR** 25th percentile **18.4 (12.9–27.7)**	–	–
Results	MTDD Median months (95% BI)		8.3 (7.4–11.1)	–	–	NR-NR 25th percentile: 5.62 (4.63–7.39)	–	NR-NR 25th percentile: 3.7 (2.8–4.6)	Results in figure only		–	–
	P value^a^		**P** = **0.032**^b^			25th percentile **P < 0.0001**^b^		25th percentile **P < 0.0001**^b^		25th percentile **P < 0.0001**^b^		
**CHAARTED**												
BL	Mean (sd)	C	**119.4 ± 20.1**	**58.2 ± 6.1**	**41.9 ± 9.7**	–	–	**1.5 ± 2.1**	–	–	–	–
			Mean (se)	Mean (se)	Mean (se)	–	–	Mean (sd):	–	–	–	–
3 mo.			116.6 ± 1.1	54.1 ± 8.7	36.1 ± 11.3			1.6 ± 2.2			–	–
6 mo.			118.4 ± 1.1	52.7 ± 9.7	39.4 ± 10.0			2.0 ± 2.4			–	–
9 mo.			118.4 ± 1.2	53.7 ± 8.5	39.6 ± 10.3			2.0 ± 2.3			–	–
12 mo.			119.2 ± 1.3	53.6 ± 8.6	39.4 ± 10.7			1.9 ± 2.3			–	–
BL	Mean (sd)	E	**118.7 ± 22.1**	**57.4 ± 7.4**	**40.9 ± 10.7**			**1.6 ± 2.3**	–	–	–	–
			Mean (se)	Mean (se)	Mean (se)	–	–	Mean (sd):	–	–	–	–
3 mo.			118.3 ± 1.2	56.4 ± 8.1	40.4 ± 10.8			1.8 ± 2.3				
6 mo.			116.7 ± 1.3	56.1 ± 7.8	40.2 ± 10.4			1.8 ± 2.4				
9 mo.			117.5 ± 1.3	55.4 ± 8.6	40.1 ± 10.7			1.8 ± 2.4				
12 mo.			116.4 ± 1.3	54.9 ± 8.4	38.4 ± 11.2			1.9 ± 2.2				
3 mo. 6 mo. 12 mo	P value^a^		**P** = **0.02**^b^ **P** = **0.04**^b^	**P < 0.0001**^b^ **P < 0.0001**^b^ **P** = **0.0300**^b^	**P < 0.0001**^b^ **P** = **0.1400** **P** = **0.3200**	–	–	No significant P values found	–	–	–	–
**TITAN**												
BL	Mean (sd)	C	**112.76** (20.16)	–	–	**1.93 ± 2.19**	–	–	Data in a Fig. 5: EQ-5D-5L VAS + EQ-5D-5L health utility index were maintained and similar between treatment arms	**2.04** (2.17)	–	–
Results	MTTD		9.23 mo.	–	–	19.09 mo.	–	–		NR	–	–
BL	Mean (sd)	E	**111.49** (19.44)	–	–	**1.84 ± 2.13**	–	–		**2.08** (2.20)	–	–
Results	MTTD		8.87 mo.	–	–	11.99 mo.	–	–		NR	–	–
	P value^a^		P = 0.85			P = 0.20						
**ARCHES**												
BL	Mean (sd)	C	**113.86** (19.82)	–	–	**1.80 ± 2.40**	**1.36 ± 1.81**	**1.33 ± 2.02**	**74.43 ± 17.14**	Only time to confirmed, clinically meaningful deterioration of bowel symptoms barely significant, favouring ADT + ENZ (Fig. 2)	–	Subscores only
Results wk 73	LSM (se)		−3.17 (1.30)	–	–	0.54 ± 0.19	0.49 ± 0.15	0.71 ± 0.15	0.28 ± 1.16		–	Subscores only
BL	Mean (sd)	E	**112.69** (18.96)	–	–	**1.77 ± 2.30**	**1.35 ± 1.68**	**1.27 ± 1.87**	**74.19 ± 16.68**		–	Subscores only
Results wk 73	LSM (se)		−1.71 (1.42)	–	–	0.33 ± 0.20	0.38 ± 0.16	0.58 ± 0.17	0.19 ± 1.27		–	Subscores only (Fig. 2)
	P value^a^		No significant difference in FACT-P total Physical wellbeing scale, **P** = **0.024**^b^ C favours over E in LV disease	–	–	No significant differences	No significant differences					
**HORRAD**												
BL	Mean (sd)	C	–	–	–	–	–	–	–	–	**70.2 ± 22.8**	Urinary symptoms significantly worse at 3 month, and for bowel symptoms at 3, 9, 12 and 24 months in the ADT + EBRT arm compared to ADT alone
3 mo.			–	–	–	–	–	–	–	–	77.8 ± 17.2	
6 mo.			–	–	–	–	–	–	–	–	76.2 ± 17.4	
12 mo.			–	–	–	–	–	–	–	–	75.6 ± 19.1	
24 mo.			–	–	–	–	–	–	–	–	79.2 ± 17.8	
BL	Mean (sd)	E	–	–	–	–	–	–	–	–	**70.0 ± 22.6**	
3 mo. 6 mo.			–	–	–	–	–	–	–	–	76.1 ± 19.4 73.5 ± 20.4	
12 mo.			–	–	–	–	–	–	–	–	74.2 ± 19.5	
24 mo.			–	–	–	–	–	–	–	–	76.6 ± 19.2	
P value	P value^a^ diff.											
3 mo.											P = 0.4	
6 mo.											P = 0.4	
12 mo.											P = 0.7	
24 mo.											P = 0.3	
**STAMPEDE DOC versus AAP**												
BL	Mean (sd)	C									**76.1 ± 19.3**	
3 mo.											73.8 ± 21.9	
6 mo.											72.1 ± 22.4	
12 mo.											71.5 ± 21.0	
24 mo.											72.6 ± 21.0	
BL	Mean (sd)	E									**77.8 ± 20.0**	
3 mo.											70.7 ± 22.2	
6 mo.											67.9 ± 21.1	
12 mo.											74.4 ± 20.2	
24 mo.											72.6 ± 20.7	
BL	Mean (sd)	E2									**78.0 ± 19.3**	
3 mo.											76.5 ± 19.5	
6 mo.											75.6 ± 20.9	
12 mo.											75.75 ± 20.4	
24 mo.											77.3 ± 17.8	
P values	P value^a^ diff.											
3 mo.											**P** = **0.001**^b^	
6 mo.											**P** < **0.001**^b^	
12 mo.											**P** = **0.412**	
24 mo.											**P** = **0.048**^b^	
**STAMPEDE arm H**											EORTC-QLQ-C30. Model-estimated Global QoL^###^: Difference in weighted average: −0.8% (95% CI −2.5% to 0.9%; P = 0.349); Model-esti-mated QLQ-30 Summary Score^###^ Difference in weighted average: −1.2% (95% CI −2.4% to 0.0%; P = 0.050).	
**ENZAMET**												
BL	Mean (sd)	C	–	–	–	–	–	–	∗∗5D-5L will be reported separately as part of a health economic evaluation.	–	**76.7 (18.5)**	Data points of PR25 results are provided in Figures
3 mo.	Mean (sd)		–	–	–	–	–	–		–	71.8 (19.1)	
6 mo.			–	–	–	–	–	–		–	73.0 (18.4)	
12 mo.			–	–	–	–	–	–		–	75.6 (17.4)	
24 mo.			–	–	–	–	–	–		–	75.6 (18.1)	
BL	Mean (sd)	E	–	–	–	–	–	–		–	**75.9 (18.9)**	
3 mo.			–	–	–	–	–	–		–	69.2 (19.8)	
6 mo.			–	–	–	–	–	–		–	71.5 (19.5)	
12 mo.			–	–	–	–	–	–		–	72.2 (18.3)	
24 mo.			–	–	–	–	–	–		–	73.6 (18.2)	
	P value^a^ overall difference		–	–	–	–	–	–		–	−1.2 (0.8) **P** = **0.10**	

Table 2 provides an overview of studies incorporating different QoL tools in mHSPC phase III RCTs, illustrating that the differences in measures and differences in ways of collecting, analysing and reporting precludes any cross-comparison that would allow to support clinical decision making.

C, Control group; E, Experimental group; BL, Baseline; mo, months; MTTD, Median time to deterioration in months; AAP, abiraterone and predniso(lo)n; ADT, androgen deprivation therapy; DOC, docetaxel chemotherapy; ENZ, enzalutamide; LV, Low Volume disease.

###STAMPEDE arm H; EORTC-QLQ-C30. ###Model-estimated Global QoL (partly conditional analysis in all patients). Difference in weighted average: −0.8% (95% CI −2.5% to 0.9%; P = 0.349) and (composite outcome analysis in all patients). Difference in weighted average: 1.3% (95% CI −1.1% to 3.8%; P = 0.287). ###Model-estimated QLQ-30 Summary Score (partly conditional analysis in all patients). Difference in weighted average: −1.2% (95% CI −2.4% to 0.0%; P = 0.050). (partly conditional analysis in all patients). Cross-sectional analyses of both Global QoL and QLQ-30 Summary Score: poorer QoL at week 12 after randomisation for patients allocated to SOC + RT—Global QoL absolute difference −2.9% (95% CI −4.8% to −1.0%, P = 0.003); Summary Score absolute difference −2.0% (95% CI −3.2% to −0.8%, P = 0.001)—but not at other assessments.

a All P values in the Table are taken directly from the individual studies; P values are depicted in bold.

b Time points with significant difference in QoL.

c FACT-P see also Table 3. FACT-P Total, FACT-P Physical scale and FACT-P TOI.

d BFI: i.e. worst fatigue.

**Table 3:** FACT-P|Functional assessment of cancer therapy–prostate outcomes in 4 RCTs.

	FACT-P	Total								Physical scale								TOI							
		CG				EG				CG				EG				CG				EG			
LATITUDE∗																									
Baseline	Mean (sd)	112.4 (20.0)				113.2 (20.0)				23.2 (4.5)				23.4 (4.6)				73.7 (15.4)				73.8 (15.3)			
	P value	–								–								–							
Results	Median time to deterioration (mo) (95% CI) *(MTTD)*	8.3 (7.4–11.1)				12.9 (9.0–16.0)				7.4 (6.5–9.2)				14.4 (10.2–18.2)				9.2 (7.4–11.2)				18.4 (14.4–22.6)			
	HR (95% CI), **P**^a^	0.85 (0.74–0.99), **P** = **0.032**∗								0.75 (0.65 = 0.87), **P** = **0.0001**∗								0.73 (0.63–0.85), **P** = **0.0001**∗							
CHAARTED∗∗																									
Baseline	Mean (sd)	118.7 (22.1)				119.4 (20.1)				23.7 (0.3)°				24.1 (0.2)°				77.8 (0.9)°				78.7 (0.8)°			
	P value	**P** = **0.91**								**P** = **0.39**								**P** = **0.92**							
Results	Months	3	6	9	12	3	6	9	12	**3**^∗^	6	9	12	**3**^∗^	6	9	12	**3**^∗∗^	6	9	12	**3**^∗∗^	6	9	12
	Mean	118.3	116.7	117.5	116.4	116.6	118.4	118.4	119.2	**23.0**	23.0	22.9	22.4	**21.4**	22.8	23.0	22.7	**77.6**	76.3	76.8	75.5	**74.9**	76.9	76.8	77.4
	P^a^ diff. at 3 months	**P** = **0.0700**								**P** < **0.0001**								**P** ≤ **0.0030**							
TITAN																									
Baseline	Mean (sd)	111.49 (19.436)				112.76 (20.158)				23.89 (3.880)				24.21 (3.776)				73.87 (14.137)				75.04 (13.034)			
	P value	–								–								–							
Results	Median time to deterioration (mo) (95% CI) *(MTTD)*	9.23 (7.39–12.91)				8.87 (4.70–11.10)				–				–				–							
	HR (95% CI), P^a^	1.02 (0.85–1.22), **P** = **0.85**								–								–							
	HR (95% CI) of time to event analysis	1.017 (0.850–1.217)				**P value NR**				1.136 (0.952–1.357) **P value NR**								1.026 (0.853–1.234)				**P value NR**			
ARCHES																									
Baseline	Mean (sd)	112.69 (18.96)				113.86 (9.82)				23.48 (4.28)				23.52 (4.41)				74.46 (13.95)				75.65 (13.38)			
	P value^a^	–								–								–							
Results	Least squares mean (se)	−1.71 (1.42)				−3.17 (1.30)				−0.40 (0.34)				−1.42 (0.3)				−1.29 (1.07)				−3.15 (0.98)			
	**TD** (95% CI)	1.47 (−5.12, 2.18)								−1.02 (−1.90 to 0.13)								−1.88 (−4.62 to 0.87)							
	HR (95% CI), P^a^	0.89 (0.0773–1.07), **P** = **0.209**								0.95 (0.78–1.15) **P** = **0.581**								0.96 (0.79–1.17), **P** = **0.686**							

Table 3 provides an overview of results from the four RCTs which used FACT-P illustrating differences in collecting, analysing and reporting of results.

Abbreviations: sd, standard deviation; mo, months; NR, Not Reported; TD, treatment difference for enzalutamide (ENZ) versus PBO (placebo).

∗Time points with significant difference in QoL. ∗LATITUDE showing significantly more favourable time to deterioration of FACT-P total, physical scale and TOI for the investigational arm compared to the control ADT arm. ∗∗CHAARTED showing significantly worse Physical domain and TOI of FACT-P at time point 3 months.

a All P values in the Table are taken directly from the individual studies; P values are depicted in bold.

**Table 4:** BPI-SF|Pain outcomes in 4 RCTs.

	BPI SF	Worst pain (item 3)		Pain interference (scale)								Pain severity							
		CG	EG	CG				EG				CG				EG			
LATITUDE												**Item 3**–**6**							
Baseline	Mean (sd)	2.2 (2.4)	2.2 (2.5)	1.5 (2.0)				1.5 (2.0)				–				–			
	P value	–		–								–							
Results	Median time to deterioration (95% CI) (mo)	**NR (NR-NR)**	**NR (NR-NR)**	18.4 (14.5–27.7)				**NR (NR-NR)**				NR (NR-NR)				NR (NR-NR)			
	25 P_*i*_ (95% CI)	5.62 (4.63–7.39)	11.07 (9.23–18.43)	3.7 (2.8–4.6)				6.5 (4.6–9.2)				30.3 (18.7-**NR**)				**NR (NR-NR)**			
	HR (95% CI), P^a^	**0.63** (0.52–0.77),	**P < 0.0001**^b^	**0.67** (0.56–0.80), **P < 0.0001**^b^								**0.90** (0.69–1.16), **P** = **0.41**							
CHAARTED												**Average 4 items of worst, least, average and now.**							
Baseline	Mean (sd)	–	–	1.6 (2.3)				1.5 (2.1)				1.7 (1.9)				1.6			
	P value	–	–	**P** = **0.850**								**P** = **0.5000**							
Results	Time (mo)	–		3	6	9	12	3	6	9	12	3	6	9	12	3	6	9	12
	Mean score (sd)	–		1.8 (2.3)	1.8 (2.4)	1.8 (2.4)	1.9 (2.2)	1.6 (2.2)	2.0 (2.4)	2.0 (2.3)	1.9 (2.3)	1.9 (2.1)	1.8 (2.1)	1.9 (2.1)	2.0 (1.9)	1.7 (1.0)	2.0 (2.1)	2.2 (2.2)	2.0 (2.0)
	P value	–		No significant differences								No significant differences							
TITAN												**Median time to pain progression**							
Baseline	Mean (sd)	1.93 (2.190)	1.84 (2.127)	–				–				–				–			
	P value	–		–								–							
Results	Median time to 1st deterioration (95% CI) (mo) **(TTFD)**	11.99 (9.28–18.46)	19.09 (11.04-NR)	NR (NR-NR)				NR (28.58-NR)				NR (NR-NR)				NR (NR-NR)			
	25 P_*i*_ (95% CI)			6.24 (4.63–7.43)				9.17 (5.55–1.96)				14.78 (11.07–19.81)				20.53 (16.10-NR)			
	HR (95% CI), P value^a^	**0.89** (0.75–1.06), **P** = **0.20**		**0.90** (0.73–1.1.), **P** = **0.29**								**0.83** (0.65–1.05), **P** = **0.12**							
ARCHES																			
Baseline	Mean (sd)	1.77 (2.30)	1.80 (2.40)	1.27 (1.87)				1.33 (2.02)				1.35 (1.68)				1.36 (1.81)			
	P value^a^	–		–								–							
Results	Least-squares mean wk 73 (se)	0.33 (0.20)	0.54 (0.19)	0.58 (0.17)				0.71 (0.15)				0.38 (0.16)				0.49 (0.15)			
	Median time to first clinical deterioration (mo) (**TTFD)**	11.10	14.09	11.14				11.24				16.76				19.38			
	HR of time to 1st clinical deterioration (95% CI), p^a^	**0.82** (0.69–0.98), **P** = **0.032**^b^		**1.00** (0.84–1.19), **P** = **1.0**								**0.79** (0.65–0.97), **P** = **0.021**							
	Median time to 1st confirmed clinical deterioration (mo) **(TTFCD**)	22.11	19.58	22.11				17.08				22.11				NR			
	HR time to 1st clinical deterioration (95% CI), P^a^	**0.82** (0.67–1.02), **P** = **0.075**		**0.95** (0.78–1.16), **P** = **0.6**								**0.85** (0.66–1.10), **P** = **0.2**							

Table 4 provides an overview of results from the four RCTs which used BPI-SF illustrating differences in collecting, analysing and reporting of pain outcome results.

Abbreviations: sd, standard deviation; mo, months; NR, Not Reported; HR, Hazard ratio; CI, Confidence Interval.

a All P values in the Table are taken directly from the individual studies; P values are depicted in bold.

b Time points with significant difference in QoL. CHAARTED showing significantly worse Physical domain and TOI of FACT-P at time point 3 months. LATITUDE showing significantly more favourable time to deterioration of FACT-P total, physical scale and TOI for the investigational arm compared to the control ADT arm.

### Risk of bias assessment

Of the eight RCTs, three studies were judged as having no serious methodological limitations and only a low Risk of Bias (Table 5). Five were judged as high Risk of Bias according to the RoB2 guidelines Version 2019^16^ and thus as having some methodological limitations.

**Table 5:** Risk of bias assessment of included RCT's on QoL in de novo mHSPC treatment.

	Random sequence generation	Allocation concealment	Blinding of participants and personnel	Incomplete outcome data	Selective reporting	Overall bias
PATCH						
LATITUDE						
CHAARTED						
TITAN						
ARCHES						
HORRAD						
STAMPEDE DOC versus ABI; Arm H (EBRT)						
ENZAMET						

Risk of bias was assessed using the Cochrane Risk of Bias for RCT guidelines V2 for each trial for Quality of Life reported outcomes.

The Cochrane Risk of Bias tool for randomized trials (RoB 2) version 2019 consists of five domains: 1) the randomization process, 2) the deviations from the intended interventions, 3) the missing outcome data, 4) the measurement of the outcome, and 5) the selection of the reported results. Each domain had up to seven questions.

The green circles represent “low risk of bias”, the yellow circles represent “some concerns”, and the red “high risk of bias”.

Main concerns were for bias based on blinding of participants and personnel since only three trials followed a double-blind design.

Three trials had missing outcome data in at least 10% of the total trial population.

Of the STAMPEDE multi-arm study, one publication (STAMPEDE, DOC versus ABI, i.e. docetaxel versus abiraterone) did not report adequately on the randomisation process and deviations from the intended intervention were unclear. The other STAMPEDE publication (arm H, ADT + EBRT versus ADT) did report these items adequately.

Only three studies (TITAN, ARCHES and LATITUDE) were considered to have low overall risk of bias.

### Quality of PRO reporting

Using the ISOQOL recommended criteria, we identified that the PRO hypothesis and specification of relevant PRO domain(s) was only provided in 2 (25%) studies, and that the rationale for choice of the PRO instrument was provided in 5 (62.5%) studies (Supplementary Table S4).

Furthermore, reasons for missing data were not explained in 4 (50%) of studies. Generalizability issues uniquely related to the PRO results were discussed in 5 (62.5%) studies. A high level of adherence to the other ISOQOL recommended standards was found.

### PRO results

Mean baseline QoL outcome in ADT standard of care arm were in general comparable between evaluable trials sharing the same QoL tools, while investigational arms showed similar QoL outcome as their controls (Table 2, Table 3, Table 4); Baseline compliance, not provided in one study, varied amongst RTCs (Table 1), in particular during follow up, with the highest overall compliance in LATITUDE (≥90% for all PRO measurement tools).

Five RCTs investigated ADT in combination with an oral ARPI (abiraterone plus prednisone, apalutamide, and enzalutamide); 2 trials used the EORTC cancer-specific questionnaire QLQ-C30 and its prostate cancer module QLQ-PR25 (+EQ-5D-5L in ENZAMET) and 3 pharma registration trials LATITUDE, TITAN and ARCHES used three instruments FACT-P, BPI-SF and EQ-5D-5L (plus BFI-SF in LATITUDE and TITAN, and plus EORTC-QLQ-PR25 in ARCHES).

For descriptive purposes, we hereunder describe PRO results by grouping RCTs based on having used either the EORTC QLQ-C30 or the FACT-P questionnaires.

### RCTs using FACT-P

RCTs LATITUDE, CHAARTED, TITAN, and ARCHES all demonstrated OS benefit, but analysed and reported FACT-P differently, namely as changes over time of FACT-P total, physical scale, and TOI, as means (CHAARTED), time to (first) deterioration (LATITUDE and TITAN) or least squares mean (ARCHES) (Tables 2 and 3).

Furthermore, pain reporting using BPI-SF was heterogeneous with different statistical analyses of pain domains, worst pain, pain interference and pain severity, amongst the 4 RCTs (Table 4).

CHAARTED^20^ investigated QoL during the first year. They showed no significant differences between treatment arms when comparing mean scores of FACT-P total, FACT-Taxane, FACIT-Fatigue and BPI (Table 2); only after applying a mixed-effects model there was a significant poorer mean FACT-P at 3 months, but better mean FACT-P at 12 months in the ADT + DOC arm. The mean changes in FACT-P at 3 and 12 months did not meet the thresholds of a clinical meaningful difference.

The three pharma-initiated registration trials investigating second generation hormonal treatment using androgen-receptor-targeted agents abiraterone (plus prednisone 5 mg daily) Fizazi et al.,^27^ and AR-inhibitors apalutamide Chi et al.^29^ or enzalutamide Armstrong et al.^30^ added to ADT versus placebo added to ADT. Primary endpoints were OS and (r)PFS per RECIST v1.1 for LATITUDE and TITAN and rPFS for ARCHES. LATITUDE had a considerably longer median follow up at the time of reporting than TITAN and ARCHES. HR for (r)PFS between the experimental treatment arm and ADT plus placebo arm were in the same range with a HR for LATITUDE of HR 0.47 95% CI 0.39–0.55, for TITAN HR 0.48 95% CI 0.39–0.60 and ARCHES HR 0.39 95% CI 0.30–0.50.

All three trials assessed median time-to (clinical or first confirmed clinical) deterioration (median time to deterioration, MTD; time to first clinical deterioration, TTCD; time to first clinically confirmed deterioration, TTFCD) of BPI-SF, median time to deterioration of FACT-P (LATITUDE and TITAN) and also EQ-VAS for ARCHES.

LATITUDE^19^ reported a statistically significant, not clinically meaningful, improved FACT-P total and a significantly longer median time to FACT-P deterioration (physical scale, TOI), and longer and clinically meaningful time to pain progression favouring the experimental arm (Tables 2 and 3). At baseline, mean worst pain was higher in LATTITUDE than in the other RCTs; median time to pain deterioration was not reached (NR) in both arms of LATITUDE at the time of QoL reporting. Time to deterioration of worst pain (HR 0.63) and pain interference (HR 0.67) was significantly delayed by abiraterone plus prednisone versus placebo (Table 4).

TITAN^21^ also reported median times to deterioration of QoL: they, however, showed no significant or clinically meaningful difference in the various PROs, including FACT-P, BPI-SF pain, BFI fatigue, and EQ-VAS (Table 2, Table 3, Table 4).

ARCHES^22^ demonstrated improved rPFS (primary endpoint) and OS benefit especially in the low-volume subgroup.^37^

They reported no statistically significant or clinically meaningful difference for average FACT-P (total, physical, TOI), BPI-SF pain intensity and interference, or EQ-VAS score changes; EORTC QLQ-PR25 subdomain analyses did not show any differences between treatment arms (Table 2).

ARCHES demonstrated a significant longer median time to first clinical deterioration of worst pain (HR 0.82) and pain severity (HR 0.79) (Table 4, TTFD). There were, however, no significant differences in BPI when time to first clinical confirmed deterioration (TTCFD) was assessed.

Between LATITUDE, TITAN and ARCHES HRs for time to deterioration for worst pain, pain interference and pain severity varied between 0.63 and 1.00, with a greater delay in pain progression in the experimental arm of LATITUDE as compared to that of the experimental arm of ARCHES compared to the control arm. Since these 3 RCTs all allowed pain medication but were run in many sites of many regions of the world varying between 202 and 235 sites in 23–34 countries (Supplementary Table S5) no firm conclusion can be drawn as to whether differences in pain medication use affected the outcome of pain progression.

Based on EQ-5D-5L patients receiving the investigational treatment in LATITUDE and ARCHES seemed to do better than those receiving ADT plus placebo, while no differences between treatment arms were observed in TITAN. EQ-5D-5L data in LATITUDE indicated better general health status scores assessed by the EQ-VAS and health utility scores in patients receiving abiraterone acetate and prednisone plus ADT than placebo plus ADT. These improvements were observed throughout the study. In TITAN results from EQ-5D-5L and EQ-5D-5L visual analogue scale scores were similar between the two treatment groups, with EQ-5D-5L health utility index declining while EQ-5D-5L visual analogue scale scores were maintained over time. In ARCHES the median time to deterioration on EQ-5D-5 L VAS was significantly delayed with enzalutamide plus ADT versus placebo plus ADT (TTFD 11.14 versus 8.38 month; HR 0.80, 95% CI 0.67–0.94; nominal P = 0.0070). TTD for first clinical confirmed deterioration (TTFCD) for EQ-5D-5 L VAS still showed a significant between-group difference in favour of enzalutamide.

### RCTs using EORTC QLQ-C30

Of the RCTs PATCH, HORRAD, STAMPEDE, ENZAMET the latter two trials demonstrated OS benefit. PATCH investigators^18^ only reported on their 6 months global QoL from the phase 2 and 3 together with better overall global QoL in investigational tE2 compared to control ADT (Table 1, Table 2). HORRAD^23^ reported significantly and clinically meaningful worse, but mostly temporarily, urinary and bowel symptoms in EORTC QLQ-PR25 in the investigational ADT + EBRT arm. In 22% of patients, bowel symptoms remained after 24 months; general HRQoL did not deteriorate (Table 2). STAMPEDE^24^ reported a direct, randomized comparative HRQoL analysis of two investigational arms of STAMPEDE (ADT + DOC + prednisolone 5 mg daily during the 6 courses of docetaxel treatment^32^ and ADT + AAP + prednisolone 5 mg daily) previously shown to improve OS compared to ADT.^33^^,^^34^

Average EORTC QLQ-C30 (Q29 & 30) global scores were significantly and clinically meaningful lower with ADT + DOC at 3 and 6 months compared to that with ADT + AAP. Longitudinal analysis over the 2-year period after random assignment showed that mean modelled EORTC-QLQ-C30 (Q29 & 30) global-QOL score was significantly higher in ADT + AAP, the predefined threshold for clinical significance. Over 2 years, QLQ-C30 symptoms fatigue and pain favoured the investigational arm, but this was only clinically meaningful for pain scores. Of the QLQ-C30 functional domains only social functioning scores reached the clinically meaningful difference threshold in favour of ADT + AAP in repeated measures analyses over 24 months.

STAMPEDE trial also reported long-term results of ADT plus radiotherapy added to the prostate versus ADT (SOC) and found no evidence of a difference in QoL scores in the first 2 years.^25^ Data were reported as absolute difference of average Global QoL and average QLQ-30 Summary Score across all patients, next to cross-sectional analyses of both Global QoL and QLQ-30 Summary Score at week 12.

ENZAMET investigators^26^ reported worsening of self-reported fatigue, cognitive function, and physical function in the enzalutamide treatment arm, but not of Global Health Status/Qol (Q29 & 30) (Table 2). The observed differences in QoL between the 2 treatment arms did not fulfil the prespecified requirements for minimum clinically important differences.

## Discussion

This study demonstrates the methodological heterogeneity in HRQoL studies (e.g. differences in HRQoL measures. individual endpoints, collection time points, and statistical methods used to collect and analyse PRO data) and the risk of bias (lack of blinding of participants, absence of a priori hypothesis and methods addressing missing data) precluding solid cross-comparison of HRQoL trial data and decision-making despite apparent HRQoL homogeneity between patient groups.

Some studies did not provide a definition of statistical and clinical meaningful changes in PRO outcomes, while the majority did not report a PRO hypothesis.

Most RCTs failed to show significant differences in QoL between treatment arms; some studies reported significant but no clinically meaningful changes, few studies showed significant and clinically meaningful differences between treatment arms. A post hoc analysis of the TITAN study assessing apalutamide treatment effect on HRQoL by patient age: <65, 65–79 and ≥ 80, or <75 and ≥ 75 years showed similar HRQoL across treatment and age groups.^38^

HRQoL were not primary endpoints in these trials and in the absence of a statistical power analysis for HRQoL outcome measures, we cannot rule out the possibility that a failure to detect a difference, HRQoL outcomes might reflect a lack of statistical power rather than an actual lack of effect.

Although these contemporary trials were seemingly executed in a unique stage of metastatic prostate cancer defined as metastatic hormone-sensitive disease, there was considerable variation in patient populations with regard to presence of unfavourable prognostic factors such as disease burden (high volume disease), aggressive biology of the disease (high risk [HR] disease, synchronous metastatic disease (de novo metastatic disease). For instance, LATITUDE consisted of 100% de novo mHSPC patients, while the TITAN, ARCHES, and ENZAMET trials included 81%, 67%, and 67% of these patients, respectively. mHSPC trials also differed with respect to inclusion of patients with non-metastatic disease (e.g. STAMPEDE trial), allowing prior systemic treatment (prior docetaxel treatment, prior ADT), and per-protocol differences in allowing cross-over in the control arm to the investigational treatment after progression.

In addition, there were differences in sample size, duration of follow-up, number of participating sites, countries and geographical regions (Supplementary Table S5), especially between pharma-sponsored and investigator-initiated trials. This is particularly important as differences in guidelines or availability of co-medications, and cross-cultural differences in coping with incurable disease, may also affect HRQoL. Clinical trials should preferably include participants from diverse regions and cultural backgrounds to gather data on how different populations respond to treatments.

Concomitant prolonged use of a low doses of prednisone 5 mg daily with abiraterone treatment in LATITUDE and STAMPEDE, are of note as prednisone has been shown to mediate modest therapeutic benefit in mCRPC patients.^39^, ^40^, ^41^, ^42^ Corticosteroids are also known for their pleiotropic, dose- and duration-related adverse effects including bone loss, immunosuppression, hyperglycaemia and aggravating diabetes, mood and cognitive alterations. The long-term prednisone administration with abiraterone, but potentially also the shorter period of a higher dose of prednisolone during docetaxel treatment in STAMPEDE may have affected HRQoL.

Financial constraints may prevent long-term AE and HRQoL data collection, leading to underreporting of AEs and their impact on patient outcomes. It is crucial that healthcare providers and patients be vigilant and report these long-term adverse events through post-marketing surveillance.

The use of time to deterioration (TTD) in three registration trials investigating addition of oral hormonal treatment added to ADT versus ADT plus placebo is of interest, given its similarity to progression-free survival. TTD of HRQoL may provide clinical information which is informative to cancer patients and clinicians. In this respect differences in HR for TTD of pain and FACT-P between LATITUDE, TITAN and ARCHES suggest that abiraterone plus prednisone is better than apalutamide and enzalutamide in delaying time to pain progression, and better than apalutamide in delaying time to progression in FACT-P.

Intense pain is a debilitating symptom that frequently occurs in prostate cancer patients as 80% of metastatic patients develop skeletal metastases with an increased risk of skeletal-related events such as fractures and spinal cord compression. Time to pain progression is therefore an attractive endpoint that may be compared to clinically relevant time-to-event efficacy outcomes such as radiographic progression. The 3 trials reporting on time to pain progression, LATITUDE, TITAN and ARCHES, had many features in common: they investigated the addition of an oral ARPI to ADT, were the only placebo-controlled and double blinded RCTs with a low risk of bias assessed based on the RoB2 risk of bias tool, executed on a global scale in many countries and continents in a relatively short period of time, and sharing 3 HRQoL tools including the brief pain inventory short form tool (BPI-SF). They performed longitudinal HRQoL assessments over a similar long period of time, till end of treatment (LATITUDE) or disease progression (TITAN and ARCHES), enabling comparison of their time to pain progression and HRQoL worsening outcomes. Despite these similarities in the absence of a formal randomized trial comparing these 3 ARPIs each combined with ADT no definitive conclusions can be drawn based on the much lower hazard ratio for TTD of BPI-SF obtained in LATITUDE compared to that in TITAN or ARCHES as to whether abiraterone plus prednisone plus ADT is better in delaying pain compared to apalutamide plus ADT or enzalutamide plus ADT.

In future trials HRQoL worsening and time to pain progression may be further explored by systematic assessments of time to HRQoL worsening and comparison with conventional radiological methods to monitoring disease progression.

The transient QoL decline at 3–6 months of patients assigned to ADT plus docetaxel chemotherapy in CHAARTED (using FACT-P) and STAMPEDE (using EORTC-QLQ-C30), are consistent with earlier observations in the GETUG-AFU-15 trial (using EORTC-QLQ-C30),^43^ and coincide with known acute side effects of chemotherapy. Clinicians and patients thus need to weigh the transient chemotherapy-induced QoL decline at 3 months, patient's co-morbidity and preference, to the OS benefit of the chemotherapy-ADT combination.

Two trials ARASENS^44^ and (not completed) PEACE-1^45^ comparing a triplet combination to the doublet of ADT plus docetaxel have also shown improved clinical outcome (Supplementary Table S6). Although the ARASENS trial has a major impact on the community's opinion with regard to standard of care its HRQoL outcome their HRQoL outcome has not been published as a peer-reviewed publication although their clinical outcome was published 2.5 years ago in February 2022.

Importantly, in the five trials investigating the addition of ARPIs to ADT in mHSPC, different HRQoL instruments or combinations of instruments were used. Also, different definitions of endpoint assessments and methods of analysis for the same HRQoL instrument were used obscuring the ability for cross-comparison of HRQoL results between trials. HRQoL data in STAMPEDE Arm H were provided as difference, using mean estimates, precluding comparison with other RCTs using EORTC QLQ C30. The HORRAD study, which also investigated the effect of adding radiotherapy to the prostate, reported prostate-specific EORTC-QLQ-PR25 data in addition to EORTC QLQ-C30.

There are a number of tools to assess Risk of Bias in RCTs^16^^,^^46^^,^^47^ of which RoB2^16^ is still considered one of the most comprehensive and reliable tools for assessing the risk of bias in randomized trials, whereas another tool (e.g. JADAD tool^47^) is not time consuming, relatively simple but neglects some important sources of bias. In our study, we used the validated and widely used RoB2 tool.^16^ Only three publications (LATITUDE, TITAN and ARCHES) were considered to have low overall risk of bias. An evaluation of publication bias (e.g. through a funnel plot), was not feasible due to the use of different HRQoL tools across trials and the reporting of varying selections of components of these tools.

A strength of our systematic review was the focus on contemporary large phase III randomized trials, of which the results may serve as a benchmark for QoL reporting in metastatic hormone-sensitive prostate cancer. However, our study also has limitations. Of the included RCTs published since 2015 the protocols were written longer time ago, while the application of questionnaires developed and validated decades ago may inadequately capture QoL during treatments with novel oncological agents. Also, no objective measurement of physical activity by a device was done, while QOL measures by nature are subjective in measurement in open-label randomized trials.

We did not evaluate the trial protocol and our review of HRQoL is not an evaluation of the overall quality of the QoL study design. The analysis of the quality of PRO reporting revealed that, overall, the level of adherence to the ISOQOL recommended standards in trials publications was high, confirming a trend that showed an improvement in the quality of PRO reporting in cancer RCTs over the past few years.^48^ However, it should be noted that we used both publications and clinical protocols to evaluate this aspect, and this may have increased the adherence to the ISOQOL recommended standards. Despite these promising results, there are still some areas of improvements, e.g. regarding key aspects such as the reporting of the PRO hypothesis or the reasons of missing data.

The use of similar methodologies for PRO and HRQoL assessments across different studies facilitate meaningful comparisons by following guidelines for improving the design of clinical trials including PROs.^49^, ^50^, ^51^, ^52^ This includes standardizing how results are reported and interpreted. Also, it is important to ensure transparency in analysis and reporting. For instance, through external audits or secondary/central analyses by an independent committee we can ensure credibility of the data. Key guidelines for improving the design of clinical trials including PROs^50^ and the reporting of PRO results in trial publications^49^ may help generating clinically relevant data that can help making more informed decisions. International initiatives aimed at standardizing PRO analyses in cancer RCTs are also ongoing.^51^

Most PRO tools were developed and validated longer time ago, before the emergence of novel often oral anticancer therapies with different toxicity profiles. Sensitivity of PRO instruments may not be adequate nor relevant to the condition of contemporary patients and current treatments. Therefore, standard PRO measures may indeed not be sensitive enough to detect the full range of side effects associated with novel therapies. The use of item libraries such as the PRO CTCAE (https://healthcaredelivery.cancer.gov/pro-ctcae/), the FACIT Searchable Library (https://www.facit.org/facit-searchable-library), and the EORTC Item Library (https://www.eortc.be/itemlibrary/) may offer the advantage of selecting specific items that may be more sensitive for detecting the different spectrum of potential novel side effects. An approach to increase the sensitivity of PRO measures in future studies could be, for example, to use static questionnaires in conjunction with specific items from libraries.

Finally, the current digital era provides us with the opportunity to collect quantitative data in real-time. Digital tools now allow us to collect data of the patient's activity and daily mobility level, including mobility data by monitoring vehicle driving, and hours of sleep. Longitudinal quantitative QoL data could provide relevant information that are compelling for decision-makers and the community. Longitudinal PRO data collection (baseline and follow-up assessments) may well provide insights into patients' experiences and burden of disease throughout the disease condition and treatment.^51^ Patient perspectives can be captured by medical devices/instruments leading to the development medical devices is an integral step for FDA approval. FDA guidance aims to ensure medical device regulation through patient-centered evaluations.

Medical devices can potentially offer a more user-friendly interface for patients to report their outcomes. Overall, it is important to provide patients with education and training on how to use PRO tools effectively and to get their feedback to improve the tools and methodologies.

Both FDA and EMA support the use of wearable, biosensor, and other real-world data in regulatory decision making,^53^^,^^54^ but these require comparison of technology against technical gold standard if available, validation and correlation with traditional endpoints.^55^

In line with our findings Paravathaneni et al.^56^ in their recent systematic review on the quality of data reporting and analysis of 40 trials during 15 years of patient reported outcomes in clinical trials leading to GU oncology drug approvals (February 2007–July 2022) reported a lack of standardisation. They performed a detailed analysis of 20 GU oncology, including 11 prostate cancer, randomized trials using a PRO Endpoint Analysis Score (PROEAS), a 24-point scoring scale from Setting International Standards in Analysing Patient Reported Outcomes and Quality of Life Endpoints Data Consortium (SISAQOL), to assess PRO reporting standards. The authors underscored the need for improvement in quality of design and conduct of PRO endpoint in future trials and accelerated publication of PRO endpoints, using standardized analysis, and prespecified hypothesis driven endpoints.

Abi Jaoude et al.^57^ identified 790 phase 3 cancer trials in the same registry database; only 28.4% used for oncology drug approvals by FDA till Feb. 2020 and only 0.9% of phase 3 RCTs had QoL as primary endpoint. Mandarino et al.^58^ investigated QoL reporting in 446 oncological phase III trials, published in 11 major journals in the period 2012–2016. QoL was not included as endpoint in less than half of the trials, QoL was frequently underreported, and QoL publications delayed.

Marandino et al.^59^ investigated QoL of phase III oncological drug trials in different stages of prostate cancer and published 2012–2018; A third of the trials did not include QoL as an endpoint. Moreover, QoL data were not reported in about half of the primary publications of the trials, and this did not differ between the trials in the various stages of prostate cancer. Methodology of QoL analysis was heterogeneous for type of instruments, analysis, and presentation of results.^59^

Samuel et al.^60^ highlighted publication bias. They performed a retrospective analysis of 45 phase 3 Cancer Drug RCTs published in 2019 enrolling 24,806 participants. The authors showed that only a quarter of new drugs were associated with improvement in QoL. Only 22% of trials with improvement of progression-free survival also demonstrated improved QoL. Importantly, QoL outcome was reported in a favourable way in about fifty percent of trials that did not show improvement in QoL.

The limitations identified in HRQoL publications related to contemporary mHSPC RCTs allow for only a few comments. Of the trials with OS benefit favouring the investigational arm (LATITUDE, CHAARTED, TITAN, STAMPEDE and ENZAMET), only LATITUDE investigating the addition of abiraterone plus prednisone to ADT demonstrated improvement of QoL across multiple tools used.

Effective treatment in the highly unfavourable, high-risk metastatic HSPC patient population of LATITUDE may have resulted in more profound divergence in QoL between treatment arms; possibly, off-target effects of prednisone may have further added to increased feeling of well-being in the investigational arm. Of the 3 RCTs using single-agent, oral AR-inhibitors (TITAN, ARCHES, ENZAMET). ENZAMET only demonstrated very few significant differences in EORTC-QLQ-C30 domains, whereas TITAN did not show any significant differences over a wide range of QoL domains. It is important to note that various aspects, including the time period in which the trial was conducted, the follow-up period, and differences in patient population (e.g. differences in region and inclusion criteria) across studies, make these comparisons challenging. For instance, LATITUDE consisted of only de novo mHSPC patients, while the TITAN, ARCHES, and ENZAMET trials included 81%, 67%, and 67% of these patients, respectively.

In conclusion, heterogeneity of HRQoL studies and lack of standardisation precludes statistically meaningful cross-comparison of different trial QoL data and to confidently applying PRO results to evidence-based decision-making among all stakeholders, thus failing to support clinical decision-making in mHSPC at the time when new expensive or potentially toxic combination therapies are being introduced.

As added value of HRQoL assessment is highly dependent on the rigour and uniformity of its methods to advance the field, the observed conceptual and methodological heterogeneity calls for standardisation of HRQoL in oncology trials like standardized clinical outcome (e.g. CTCAE toxicity scores and radiological definitions to report progression-free survival).

Future clinical trial QoL reporting in a methodologically qualitative and quantitative way, using accepted definitions of clinically meaningful QoL differences, may support to QoL becoming part of regulatory approvals of novel drugs in oncology and aid in clinical decision-making that best supports patient-centred care.

## Contributors

Study concept, design: SO, AvdV-B. Writing of the protocol: SO, PW. Data curation: AvdV-B, CA, JS, PW. Methodology and verification of underlying data: FE, FS, AvdV-B, CA, SO. Analysis and interpretation of data: SO, AvdV-B, CA, FE, FS. Drafting of the manuscript: SO. Writing of the manuscript: SO, AvdV-B, CA, FE, FS, AC, SBvWvD-K. Study supervision: SO. All authors had full access to the raw data reported in this study, were involved in critical revision of the manuscript, and approved the final version submitted for publication.

## Data sharing statement

All data derived from this study are in the article. Qualified researchers may submit appropriate proposals for further inquiry to the corresponding author who will provide additional data upon specific request. Data will be available until 2 years after the publication of the study.

## Declaration of interests

FE received funding from Daiichi Sankyo (Institution) and had consultancy or advisory roles for Abbvie, Incyte, Syros, Novartis, and JAZZ Pharmaceuticals; all outside the submitted work. SBvWvD-K has grant funding from EU Cancer Mission (PRIME-ROSE, a European precision cancer medicine trial network and implementation initiative), grant no. 101104269; payment to institution (Leiden University Medical Center). All other authors declare no competing interests.

## References

[1] Cherny N.I., Sullivan R., Dafni U. (2015). A standardised, generic, validated approach to stratify the magnitude of clinical benefit that can be anticipated from anti-cancer therapies: the European Society for Medical Oncology Magnitude of Clinical Benefit Scale (ESMO-MCBS). Ann Oncol.

[2] Cherny N.I., Dafni U., Bogaerts J. (2017). ESMO-magnitude of clinical benefit scale version 1.1. Ann Oncol.

[3] Schnipper L.E., Davidson N.E., Wollins D.S. (2015). American society of clinical oncology statement: a conceptual framework to assess the value of cancer treatment options. J Clin Oncol.

[4] Schnipper L.E., Davidson N.E., Wollins D.S. (2016). Updating the American society of clinical oncology value framework: revisions and reflections in response to comments received. J Clin Oncol.

[5] FDA US. U.S. Food and Drug Administration (2009).

[6] FDA US. U.S. Food and Drug Administration (2018).

[7] FDA US. U.S. Food and Drug Administration (2021).

[8] EMA. European Medicines Agency (2016).

[9] Oosting S.F., Barriuso J., Bottomley A. (2023). Methodological and reporting standards for quality-of-life data eligible for European society for medical oncology-magnitude of clinical benefit scale (ESMO-MCBS) credit. Ann Oncol.

[10] Riaz I.B., Naqvi S.A.A., He H. (2023). First-line systemic treatment options for metastatic castration-sensitive prostate cancer: a living systematic review and network meta-analysis. JAMA Oncol.

[11] Efficace F., Feuerstein M., Fayers P. (2014). Patient-reported outcomes in randomised controlled trials of prostate cancer: methodological quality and impact on clinical decision making. Eur Urol.

[12] Page M.J., McKenzie J.E., Bossuyt P.M. (2021). The PRISMA 2020 statement: an updated guideline for reporting systematic reviews. BMJ.

[13] Kyte D., Reeve B.B., Efficace F. (2016). International Society for Quality of Life Research commentary on the draft European Medicines Agency reflection paper on the use of patient-reported outcome (PRO) measures in oncology studies. Qual Life Res.

[14] Efficace F., Bottomley A., Osoba D. (2003). Beyond the development of health-related quality-of-life (HRQOL) measures: a checklist for evaluating HRQOL outcomes in cancer clinical trials--does HRQOL evaluation in prostate cancer research inform clinical decision making?. J Clin Oncol.

[15] Efficace F., Osoba D., Gotay C., Sprangers M., Coens C., Bottomley A. (2007). Has the quality of health-related quality of life reporting in cancer clinical trials improved over time? Towards bridging the gap with clinical decision making. Ann Oncol.

[16] Sterne J.A.C., Savović J., Page M.J. (2019). RoB 2: a revised tool for assessing risk of bias in randomised trials. Bmj.

[17] Brundage M., Blazeby J., Revicki D. (2013). Patient-reported outcomes in randomized clinical trials: development of ISOQOL reporting standards. Qual Life Res.

[18] Gilbert D.C., Duong T., Kynaston H.G. (2017). Quality-of-life outcomes from the Prostate Adenocarcinoma: TransCutaneous Hormones (PATCH) trial evaluating luteinising hormone-releasing hormone agonists versus transdermal oestradiol for androgen suppression in advanced prostate cancer. BJU Int.

[19] Chi K.N., Protheroe A., Rodríguez-Antolín A. (2018). Patient-reported outcomes following abiraterone acetate plus prednisone added to androgen deprivation therapy in patients with newly diagnosed metastatic castration-naive prostate cancer (LATITUDE): an international, randomised phase 3 trial. Lancet Oncol.

[20] Morgans A.K., Chen Y.H., Sweeney C.J. (2018). Quality of life during treatment with chemohormonal therapy: analysis of E3805 chemohormonal androgen ablation randomized trial in prostate cancer. J Clin Oncol.

[21] Agarwal N., McQuarrie K., Bjartell A. (2019). Health-related quality of life after apalutamide treatment in patients with metastatic castration-sensitive prostate cancer (TITAN): a randomised, placebo-controlled, phase 3 study. Lancet Oncol.

[22] Stenzl A., Dunshee C., De Giorgi U. (2020). Effect of enzalutamide plus androgen deprivation therapy on health-related quality of life in patients with metastatic hormone-sensitive prostate cancer: an analysis of the ARCHES randomised, placebo-controlled, phase 3 study. Eur Urol.

[23] Boevé L., Hulshof M., Verhagen P. (2021). Patient-reported quality of life in patients with primary metastatic prostate cancer treated with androgen deprivation therapy with and without concurrent radiation therapy to the prostate in a prospective randomised clinical trial; data from the HORRAD trial. Eur Urol.

[24] Rush H.L., Murphy L., Morgans A.K. (2022). Quality of life in men with prostate cancer randomly allocated to receive docetaxel or abiraterone in the STAMPEDE trial. J Clin Oncol.

[25] Parker C.C., James N.D., Brawley C.D. (2022). Radiotherapy to the prostate for men with metastatic prostate cancer in the UK and Switzerland: long-term results from the STAMPEDE randomised controlled trial. PLoS Med.

[26] Stockler M.R., Martin A.J., Davis I.D. (2022). Health-related quality of life in metastatic, hormone-sensitive prostate cancer: ENZAMET (ANZUP 1304), an international, randomized phase III trial led by ANZUP. J Clin Oncol.

[27] Fizazi K., Tran N., Fein L. (2017). Abiraterone plus prednisone in metastatic, castration-sensitive prostate cancer. N Engl J Med.

[28] Sweeney C.J., Chen Y.H., Carducci M. (2015). Chemohormonal therapy in metastatic hormone-sensitive prostate cancer. N Engl J Med.

[29] Chi K.N., Agarwal N., Bjartell A. (2019). Apalutamide for metastatic, castration-sensitive prostate cancer. N Engl J Med.

[30] Armstrong A.J., Szmulewitz R.Z., Petrylak D.P. (2019). ARCHES: a randomized, phase III study of androgen deprivation therapy with enzalutamide or placebo in men with metastatic hormone-sensitive prostate cancer. J Clin Oncol.

[31] Boevé L.M.S., Hulshof M., Vis A.N. (2019). Effect on survival of androgen deprivation therapy alone compared to androgen deprivation therapy combined with concurrent radiation therapy to the prostate in patients with primary bone metastatic prostate cancer in a prospective randomised clinical trial: data from the HORRAD trial. Eur Urol.

[32] James N.D., Sydes M.R., Clarke N.W. (2016). Addition of docetaxel, zoledronic acid, or both to first-line long-term hormone therapy in prostate cancer (STAMPEDE): survival results from an adaptive, multiarm, multistage, platform randomised controlled trial. Lancet.

[33] James N.D., de Bono J.S., Spears M.R. (2017). Abiraterone for prostate cancer not previously treated with hormone therapy. N Engl J Med.

[34] Sydes M.R., Spears M.R., Mason M.D. (2018). Adding abiraterone or docetaxel to long-term hormone therapy for prostate cancer: directly randomised data from the STAMPEDE multi-arm, multi-stage platform protocol. Ann Oncol.

[35] Parker C.C., James N.D., Brawley C.D. (2018). Radiotherapy to the primary tumour for newly diagnosed, metastatic prostate cancer (STAMPEDE): a randomised controlled phase 3 trial. Lancet.

[36] Davis I.D., Martin A.J., Stockler M.R. (2019). Enzalutamide with standard first-line therapy in metastatic prostate cancer. N Engl J Med.

[37] Armstrong A.J., Azad A.A., Iguchi T. (2022). Improved survival with enzalutamide in patients with metastatic hormone-sensitive prostate cancer. J Clin Oncol.

[38] Shen J., Chowdhury S., Agarwal N. (2024). Apalutamide efficacy, safety and wellbeing in older patients with advanced prostate cancer from Phase 3 randomised clinical studies TITAN and SPARTAN. Br J Cancer.

[39] Venkitaraman R., Thomas K., Huddart R.A., Horwich A., Dearnaley D.P., Parker C.C. (2008). Efficacy of low-dose dexamethasone in castration-refractory prostate cancer. BJU Int.

[40] Tannock I.F., Osoba D., Stockler M.R. (1996). Chemotherapy with mitoxantrone plus prednisone or prednisone alone for symptomatic hormone-resistant prostate cancer: a Canadian randomized trial with palliative end points. J Clin Oncol.

[41] de Bono J.S., Logothetis C.J., Molina A. (2011). Abiraterone and increased survival in metastatic prostate cancer. N Engl J Med.

[42] Ryan C.J., Smith M.R., de Bono J.S. (2013). Abiraterone in metastatic prostate cancer without previous chemotherapy. N Engl J Med.

[43] Gravis G., Fizazi K., Joly F. (2013). Androgen-deprivation therapy alone or with docetaxel in non-castrate metastatic prostate cancer (GETUG-AFU 15): a randomised, open-label, phase 3 trial. Lancet Oncol.

[44] Smith M.R., Hussain M., Saad F. (2022). Darolutamide and survival in metastatic, hormone-sensitive prostate cancer. N Engl J Med.

[45] Fizazi K., Foulon S., Carles J. (2022). Abiraterone plus prednisone added to androgen deprivation therapy and docetaxel in de novo metastatic castration-sensitive prostate cancer (PEACE-1): a multicentre, open-label, randomised, phase 3 study with a 2 × 2 factorial design. Lancet.

[46] Luchini C., Veronese N., Nottegar A. (2021). Assessing the quality of studies in meta-research: review/guidelines on the most important quality assessment tools. Pharm Stat.

[47] Jadad A.R., Moore R.A., Carroll D. (1996). Assessing the quality of reports of randomized clinical trials: is blinding necessary?. Control Clin Trials.

[48] Efficace F., Giesinger J.M., Cella D. (2021). Investigating trends in the quality of reporting of patient-reported outcomes in oncology over time: analysis of 631 randomized controlled trials published between 2004 and 2019. Value Health.

[49] Calvert M., Blazeby J., Altman D.G., Revicki D.A., Moher D., Brundage M.D. (2013). Reporting of patient-reported outcomes in randomized trials: the CONSORT PRO extension. JAMA.

[50] Calvert M., Kyte D., Mercieca-Bebber R. (2018). Guidelines for inclusion of patient-reported outcomes in clinical trial protocols: the SPIRIT-PRO extension. JAMA.

[51] Coens C., Pe M., Dueck A.C. (2020). International standards for the analysis of quality-of-life and patient-reported outcome endpoints in cancer randomised controlled trials: recommendations of the SISAQOL Consortium. Lancet Oncol.

[52] Piccinin C., Basch E., Bhatnagar V. (2023). Recommendations on the use of item libraries for patient-reported outcome measurement in oncology trials: findings from an international, multidisciplinary working group. Lancet Oncol.

[53] FDA US (2018).

[54] Cerreta F., Ritzhaupt A., Metcalfe T. (2020). Digital technologies for medicines: shaping a framework for success. Nat Rev Drug Discov.

[55] Kruizinga M.D., Stuurman F.E., Exadaktylos V. (2020). Development of novel, value-based, digital endpoints for clinical trials: a structured approach toward fit-for-purpose validation. Pharmacol Rev.

[56] Paravathaneni M., Safa H., Joshi V. (2024). 15 years of patient-reported outcomes in clinical trials leading to GU cancer drug approvals: a systematic review on the quality of data reporting and analysis. eClinicalMedicine.

[57] Abi Jaoude J., Kouzy R., Ghabach M. (2021). Food and Drug Administration approvals in phase 3 Cancer clinical trials. BMC Cancer.

[58] Marandino L., La Salvia A., Sonetto C. (2018). Deficiencies in health-related quality-of-life assessment and reporting: a systematic review of oncology randomized phase III trials published between 2012 and 2016. Ann Oncol.

[59] Marandino L., De Luca E., Zichi C. (2019). Quality-of-Life assessment and reporting in prostate cancer: systematic review of phase 3 trials testing anticancer drugs published between 2012 and 2018. Clin Genitourin Cancer.

[60] Samuel J.N., Booth C.M., Eisenhauer E., Brundage M., Berry S.R., Gyawali B. (2022). Association of quality-of-life outcomes in cancer drug trials with survival outcomes and drug class. JAMA Oncol.

