# Supplementary material for: Health-related quality of life outcomes in randomized controlled trials in metastatic hormone-sensitive prostate cancer: a systematic review
Source: eClinicalMedicine. 2024 Nov 13;78:102914. doi: 10.1016/j.eclinm.2024.102914 (PMC11605133; doi:10.1016/j.eclinm.2024.102914)
Supplement: Supplementary Materials [file mmc1.docx]

## **SUPPLEMENTARY MATERIALS**

**Health-Related Quality of Life outcomes in randomized controlled trials in metastatic hormone-sensitive prostate cancer: A Systematic Review**

| **Drug name** | **Year of approval** | **mHSPC study** | **Indication** | **Explanatory text** |
| --- | --- | --- | --- | --- |
| Docetaxel | 2004 |  | mCRPC |  |
| Degarelix | 2008 |  | advanced PCa | new GnRH receptor antagonist |
| Cabazitaxel | 2010 |  | mCRPC | Cabazitaxel (25 mg/m^2^ every 3 weeks) |
| Sipuleucel-T | 2010 |  | mCRPC |  |
| Denosumab | 2011 |  | nonmetastatic PCa | For men receiving ADT for nm prostate cancer |
| Abiraterone + prednisone | 2011 |  | mCRPC after chemotherapy |  |
| Abiraterone  with prednisone | 2012 |  | mCRPC without prior chemotherapy |  |
| Cabazitaxel | 2017 |  | mCRPC | Lower dose of cabazitaxel (20 mg/m^2^ every 3 weeks) in combination with prednisone for pts previously treated with a docetaxel-containing treatment regimen |
| Abiraterone + prednisone | 2018 | LATITUDE | mHSPC |  |
| Enzalutamide | 2012 |  | mCRPC |  |
| Enzalutamide | 2019 |  | nmCRPC |  |
| Apalutamide | 2019 |  | nmCRPC |  |
| Enzalutamide | 2019 | ARCHES | mHSPC |  |
| Darolutamide | 2019 |  | nmCRPC |  |
| Radium-223 | 2013 |  | mCRPC |  |
| Apalutamide | 2019 | TITAN | mHSPC |  |
| Relugolix | 2020 |  | advanced prostate cancer. | Oral GnRH receptor antagonist |
| Liquid CDx test | 2020 |  | mCRPC | Diagnostic test to identify mutations in BRCA1 and BRCA2 genes in cell free-DNA isolated from plasma from patients with mCRPC eligible for rucaparib |
| Olaparib | 2020 |  | HRR + mCRPC |  |
| Rucaparib | 2020 |  | BRCA + mCRPC |  |
| 177-Lu-PSMA | 2022 |  | mCRPC |  |
| Darolutamide + docetaxel | 2022 | ARASENS | mHSPC |  |

**Supplementary Table 1. FDA approvals for treatment of prostate cancer in the period 2004 to 2023***.*

**Legend Supplementary Table 1**. Abbreviations: ADT, androgen deprivation therapy; FDA, Food and Drug Administration; HR, hazards ratio; PCa, prostate cancer; mCRPC, castration-resistant prostate cancer; mHSPC, metastatic hormone-sensitive prostate cancer; nmCRPC, non metastatic castration-resistant prostate cancer; GnRH, gonadotropin releasing hormone. Pts Patients.

Highlighted in green are approved combination treatments in metastatic hormone-sensitive prostate cancer.

## **Supplementary Table 2**. **Search Strategy**

PubMed (<https://www.ncbi.nlm.nih.gov/pubmed/>), Embase (OVID version, <http://ovidsp.ovid.com/ovidweb.cgi?T=JS&PAGE=main&MODE=ovid&D=oemezd>), Web of Scienceb(<https://www.webofscience.com/>, Cochrane Library (<http://cochranelibrary-wiley.com/cochranelibrary/search/advanced>) and Google Scholar (<https://scholar.google.com/>) were searched to identify peer-reviewed QoL reports of phase III RCTs comparing Health Related Quality of Life (HRQoL) amongst the treatment arms in mHSPC.

| **Database** | **Strategy** |
| --- | --- |
| **PUBMED** | (("Metastatic Hormone sensitive Prostate Cancer"[tw] OR "mHSPC"[tw] OR "metastatic castration naive prostate cancer"[tw] OR "metastatic prostate cancer"[tw] OR "metastatic prostate cancers"[tw] OR "metastatic prostate carcinoma"[tw] OR "metastatic prostate carcinomas"[tw] OR "metastatic prostate neoplasm"[tw] OR "metastatic prostate tumor"[tw] OR "metastatic prostate tumors"[tw] OR "metastatic prostatic cancer"[tw] OR "metastatic prostatic carcinoma"[tw] OR "metastatic prostatic carcinomas"[tw] OR "metastatic prostatic neoplasm"[tw] OR "metastatic prostatic tumors"[tw] OR (("Prostatic Neoplasms"[mesh] OR "prostate cancer"[tw] OR "prostate cancers"[tw] OR "prostate carcinoma"[tw] OR "prostate carcinomas"[tw] OR "prostate adenocarcinoma"[tw] OR "prostate adenocarcinomas"[tw] OR "prostate neoplasm"[tw] OR "prostate neoplasms"[tw] OR "prostate tumor"[tw] OR "prostate tumors"[tw] OR "prostate tumour"[tw] OR "prostate tumours"[tw] OR "prostate malignancy"[tw] OR "prostate malignancies"[tw] OR "prostatic cancer"[tw] OR "prostatic cancers"[tw] OR "prostatic carcinoma"[tw] OR "prostatic carcinomas"[tw] OR "prostatic adenocarcinoma"[tw] OR "prostatic adenocarcinomas"[tw] OR "prostatic neoplasm"[tw] OR "prostatic neoplasms"[tw] OR "prostatic tumor"[tw] OR "prostatic tumors"[tw] OR "prostatic tumour"[tw] OR "prostatic tumours"[tw] OR "prostatic malignancy"[tw] OR "prostatic malignancies"[tw]) AND ("metastatic"[tw] OR "metasta*"[tw] OR "oligometastatic"[tw] OR "oligometasta*"[tw]))) AND ("androgen deprivation therapy"[tw] OR "androgen depriv*"[tw] OR "androgen block*"[tw] OR "ADT"[tiab] OR "Androgen Antagonists"[Mesh] OR "Androgen Antagonists"[Pharmacological Action]) AND ("Quality of Life"[mesh] OR "Quality of Life"[tw] OR "Life Quality"[tw] OR "HR-QoL"[tw] OR "HRQoL"[tw] OR "QoL"[tw] OR "PROs"[tw] OR "Patient Reported Outcome Measures"[Mesh] OR "Patient Reported Outcome"[tw] OR "Patient Reported Outcomes"[tw] OR "Patient Reported"[tw] OR "Anxiety"[mesh] OR "anxiety"[tw] OR "Depression"[mesh] OR "depression"[tw] OR "Psychological Distress"[mesh] OR "distress"[tw] OR "emotional"[tw] OR "Functional Status"[Mesh] OR "functional status"[tw] OR "health outcomes"[tw] OR "health related quality of life"[tw] OR "Health Status"[Mesh] OR "health status"[tw] OR "HRQL"[tw] OR "patient outcomes"[tw] OR "PRO"[tw] OR "psychological"[tw] OR "psychosocial"[tw] OR "sexual functioning"[tw] OR "Sexuality"[Mesh] OR "Sexual Behavior"[Mesh] OR "Social Interaction"[Mesh] OR "social functioning"[tw] OR "social wellbeing"[tw] OR "social"[tw] OR "Symptom Assessment"[Mesh] OR "symptom assessment"[tw] OR "symptom burden"[tw] OR "symptom distress"[tw] OR "Fatigue"[mesh] OR "fatigue"[tw] OR "Pain"[mesh] OR "pain"[tw]) AND ("randomized controlled trial"[pt] OR "randomized"[ti] OR "randomised"[ti] OR "RCT"[ti] OR "trial"[ti] OR "Clinical Trial, Phase III"[pt] OR "phase iii"[tw] OR "phase three"[tw] OR "phase 3"[tw] OR "phaseiii"[tw] OR "phasethree"[tw] OR "phase3"[tw]) NOT "review"[pt] AND english[la] AND ("2015/01/01"[PDAT] : "3000/12/31"[PDAT]) NOT (("Clinical Trial, Phase II"[pt] OR "phase ii"[ti] OR "phase two"[ti] OR "phase 2"[ti] OR "phaseii"[ti] OR "phasetwo"[ti] OR "phase2"[ti]) NOT ("Clinical Trial, Phase III"[pt] OR "phase iii"[tw] OR "phase three"[tw] OR "phase 3"[tw] OR "phaseiii"[tw] OR "phasethree"[tw] OR "phase3"[tw]))) |
| **Embase (OVID-version)** | (("Metastatic Hormone sensitive Prostate Cancer".ti,ab OR "mHSPC".ti,ab OR "metastatic castration naive prostate cancer".ti,ab OR "metastatic prostate cancer".ti,ab OR "metastatic prostate cancers".ti,ab OR "metastatic prostate carcinoma".ti,ab OR "metastatic prostate carcinomas".ti,ab OR "metastatic prostate neoplasm".ti,ab OR "metastatic prostate tumor".ti,ab OR "metastatic prostate tumors".ti,ab OR "metastatic prostatic cancer".ti,ab OR "metastatic prostatic carcinoma".ti,ab OR "metastatic prostatic carcinomas".ti,ab OR "metastatic prostatic neoplasm".ti,ab OR "metastatic prostatic tumors".ti,ab OR ((exp *"Prostatic Cancer"/ OR "prostate cancer".ti,ab OR "prostate cancers".ti,ab OR "prostate carcinoma".ti,ab OR "prostate carcinomas".ti,ab OR "prostate adenocarcinoma".ti,ab OR "prostate adenocarcinomas".ti,ab OR "prostate neoplasm".ti,ab OR "prostate neoplasms".ti,ab OR "prostate tumor".ti,ab OR "prostate tumors".ti,ab OR "prostate tumour".ti,ab OR "prostate tumours".ti,ab OR "prostate malignancy".ti,ab OR "prostate malignancies".ti,ab OR "prostatic cancer".ti,ab OR "prostatic cancers".ti,ab OR "prostatic carcinoma".ti,ab OR "prostatic carcinomas".ti,ab OR "prostatic adenocarcinoma".ti,ab OR "prostatic adenocarcinomas".ti,ab OR "prostatic neoplasm".ti,ab OR "prostatic neoplasms".ti,ab OR "prostatic tumor".ti,ab OR "prostatic tumors".ti,ab OR "prostatic tumour".ti,ab OR "prostatic tumours".ti,ab OR "prostatic malignancy".ti,ab OR "prostatic malignancies".ti,ab) ADJ4 ("metastatic".ti,ab OR "metasta*".ti,ab OR "oligometastatic".ti,ab OR "oligometasta*".ti,ab))) AND ("androgen deprivation therapy".ti,ab OR "androgen depriv*".ti,ab OR "androgen block*".ti,ab OR "ADT".ti,ab OR "Androgen deprivation therapy"/ OR exp *"antiandrogen"/) AND (exp *"Quality of Life"/ OR "Quality of Life".ti,ab OR "Life Quality".ti,ab OR "HR-QoL".ti,ab OR "HRQoL".ti,ab OR "QoL".ti,ab OR "PROs".ti,ab OR exp *"Patient-Reported Outcome"/ OR "Patient Reported Outcome".ti,ab OR "Patient Reported Outcomes".ti,ab OR "Patient Reported".ti,ab OR exp *"Anxiety"/ OR "anxiety".ti,ab OR exp *"Depression"/ OR "depression".ti,ab OR exp *"Distress Syndrome"/ OR "distress".ti,ab OR "emotional".ti,ab OR exp *"Functional Status"/ OR "functional status".ti,ab OR "health outcomes".ti,ab OR "health related quality of life".ti,ab OR exp *"Health Status"/ OR "health status".ti,ab OR "HRQL".ti,ab OR "patient outcomes".ti,ab OR "PRO".ti,ab OR "psychological".ti,ab OR "psychosocial".ti,ab OR "sexual functioning".ti,ab OR exp *"Sexuality"/ OR exp *"Sexual behavior"/ OR exp *"Social Interaction"/ OR "social functioning".ti,ab OR "social wellbeing".ti,ab OR "social".ti,ab OR exp *"Symptom Assessment"/ OR "symptom assessment".ti,ab OR "symptom burden".ti,ab OR "symptom distress".ti,ab OR exp *"Fatigue"/ OR "fatigue".ti,ab OR exp *"Pain"/ OR "pain".ti,ab) AND (exp "randomized controlled trial"/ OR "randomized".ti OR "randomised".ti OR "RCT".ti OR "trial".ti OR *"clinical trial"/ OR "phase 3 clinical trial"/ OR "phase iii".mp OR "phase three".mp OR "phase 3".mp OR "phaseiii".mp OR "phasethree".mp OR "phase3".mp) NOT exp "review"/ AND english.la AND (2015 OR 2016 OR 2017 OR 2018 OR 2019 OR 2020 OR 2021 OR 2022 OR 2023).yr NOT (conference review or conference abstract).pt NOT (("phase 2 clinical trial"/ OR "phase ii".ti OR "phase two".ti OR "phase 2".ti OR "phaseii".ti OR "phasetwo".ti OR "phase2".ti) NOT ("phase 3 clinical trial"/ OR "phase iii".mp OR "phase three".mp OR "phase 3".mp OR "phaseiii".mp OR "phasethree".mp OR "phase3".mp))) |
| **Web of Science** | ((TI=("Metastatic Hormone sensitive Prostate Cancer" OR "mHSPC" OR "metastatic castration naive prostate cancer" OR "metastatic prostate cancer" OR "metastatic prostate cancers" OR "metastatic prostate carcinoma" OR "metastatic prostate carcinomas" OR "metastatic prostate neoplasm" OR "metastatic prostate tumor" OR "metastatic prostate tumors" OR "metastatic prostatic cancer" OR "metastatic prostatic carcinoma" OR "metastatic prostatic carcinomas" OR "metastatic prostatic neoplasm" OR "metastatic prostatic tumors" OR (("Prostatic Cancer" OR "prostate cancer" OR "prostate cancers" OR "prostate carcinoma" OR "prostate carcinomas" OR "prostate adenocarcinoma" OR "prostate adenocarcinomas" OR "prostate neoplasm" OR "prostate neoplasms" OR "prostate tumor" OR "prostate tumors" OR "prostate tumour" OR "prostate tumours" OR "prostate malignancy" OR "prostate malignancies" OR "prostatic cancer" OR "prostatic cancers" OR "prostatic carcinoma" OR "prostatic carcinomas" OR "prostatic adenocarcinoma" OR "prostatic adenocarcinomas" OR "prostatic neoplasm" OR "prostatic neoplasms" OR "prostatic tumor" OR "prostatic tumors" OR "prostatic tumour" OR "prostatic tumours" OR "prostatic malignancy" OR "prostatic malignancies") AND "metastatic")) OR AB=("Metastatic Hormone sensitive Prostate Cancer" OR "metastatic castration naive prostate cancer" OR "metastatic prostate cancer" OR "metastatic prostate cancers" OR "metastatic prostate carcinoma" OR "metastatic prostate carcinomas" OR "metastatic prostate neoplasm" OR "metastatic prostate tumor" OR "metastatic prostate tumors" OR "metastatic prostatic cancer" OR "metastatic prostatic carcinoma" OR "metastatic prostatic carcinomas" OR "metastatic prostatic neoplasm" OR "metastatic prostatic tumors" OR (("Prostatic Cancer" OR "prostate cancer" OR "prostate cancers" OR "prostate carcinoma" OR "prostate carcinomas" OR "prostate adenocarcinoma" OR "prostate adenocarcinomas" OR "prostate neoplasm" OR "prostate neoplasms" OR "prostate tumor" OR "prostate tumors" OR "prostate tumour" OR "prostate tumours" OR "prostate malignancy" OR "prostate malignancies" OR "prostatic cancer" OR "prostatic cancers" OR "prostatic carcinoma" OR "prostatic carcinomas" OR "prostatic adenocarcinoma" OR "prostatic adenocarcinomas" OR "prostatic neoplasm" OR "prostatic neoplasms" OR "prostatic tumor" OR "prostatic tumors" OR "prostatic tumour" OR "prostatic tumours" OR "prostatic malignancy" OR "prostatic malignancies") NEAR/5 ("metastatic" OR "metasta*" OR "oligometastatic" OR "oligometasta*")))) AND (TI=("androgen deprivation therapy" OR "androgen depriv*" OR "androgen block*" OR "ADT" OR "Androgen deprivation therapy" OR "antiandrogen") OR AB=("androgen deprivation therapy" OR "androgen depriv*" OR "androgen block*" OR "ADT" OR "Androgen deprivation therapy" OR "antiandrogen")) AND TS=("Quality of Life" OR "Quality of Life" OR "Life Quality" OR "HR-QoL" OR "HRQoL" OR "QoL" OR "PROs" OR "Patient-Reported Outcome" OR "Patient Reported Outcome" OR "Patient Reported Outcomes" OR "Patient Reported" OR "Anxiety" OR "anxiety" OR "Depression" OR "depression" OR "Distress Syndrome" OR "distress" OR "emotional" OR "Functional Status" OR "functional status" OR "health outcomes" OR "health related quality of life" OR "Health Status" OR "health status" OR "HRQL" OR "patient outcomes" OR "PRO" OR "psychological" OR "psychosocial" OR "sexual functioning" OR "Sexuality" OR "Sexual behavior" OR "Social Interaction" OR "social functioning" OR "social wellbeing" OR "social" OR "Symptom Assessment" OR "symptom assessment" OR "symptom burden" OR "symptom distress" OR "Fatigue" OR "fatigue" OR "Pain" OR "pain") AND TI=("randomized controlled trial" OR "controlled clinical trial" OR "randomized" OR "randomised" OR "RCT" OR "trial" OR "phase 3 clinical trial" OR "phase iii" OR "phase three" OR "phase 3" OR "phaseiii" OR "phasethree" OR "phase3") NOT DT=review AND la=english AND py=(2015 OR 2016 OR 2017 OR 2018 OR 2019 OR 2020 OR 2021 OR 2022 OR 2023) NOT dt=(meeting abstract) NOT (TI=("phase 2 clinical trial"/ OR "phase ii" OR "phase two" OR "phase 2" OR "phaseii" OR "phasetwo" OR "phase2") NOT TS=("phase 3 clinical trial"/ OR "phase iii" OR "phase three" OR "phase 3" OR "phaseiii" OR "phasethree" OR "phase3"))) |
| **Cochrane Library** | ((("Metastatic Hormone sensitive Prostate Cancer" OR "mHSPC" OR "metastatic castration naive prostate cancer" OR "metastatic prostate cancer" OR "metastatic prostate cancers" OR "metastatic prostate carcinoma" OR "metastatic prostate carcinomas" OR "metastatic prostate neoplasm" OR "metastatic prostate tumor" OR "metastatic prostate tumors" OR "metastatic prostatic cancer" OR "metastatic prostatic carcinoma" OR "metastatic prostatic carcinomas" OR "metastatic prostatic neoplasm" OR "metastatic prostatic tumors" OR (("Prostatic Cancer" OR "prostate cancer" OR "prostate cancers" OR "prostate carcinoma" OR "prostate carcinomas" OR "prostate adenocarcinoma" OR "prostate adenocarcinomas" OR "prostate neoplasm" OR "prostate neoplasms" OR "prostate tumor" OR "prostate tumors" OR "prostate tumour" OR "prostate tumours" OR "prostate malignancy" OR "prostate malignancies" OR "prostatic cancer" OR "prostatic cancers" OR "prostatic carcinoma" OR "prostatic carcinomas" OR "prostatic adenocarcinoma" OR "prostatic adenocarcinomas" OR "prostatic neoplasm" OR "prostatic neoplasms" OR "prostatic tumor" OR "prostatic tumors" OR "prostatic tumour" OR "prostatic tumours" OR "prostatic malignancy" OR "prostatic malignancies") AND ("metastatic" OR "oligometastatic"))):ti AND ("androgen deprivation therapy" OR "androgen depriv*" OR "androgen block*" OR "ADT" OR "Androgen deprivation therapy" OR "antiandrogen" OR "anti androgen" OR "antiandrogens" OR "anti androgens"):ti,ab,kw AND ("Quality of Life" OR "Quality of Life" OR "Life Quality" OR "HR QoL" OR "HRQoL" OR "QoL" OR "Patient Reported Outcome" OR "Patient Reported Outcome" OR "Patient Reported Outcomes" OR "Patient Reported" OR "Anxiety" OR "anxiety" OR "Depression" OR "depression" OR "Distress Syndrome" OR "distress" OR "emotional" OR "Functional Status" OR "functional status" OR "health outcomes" OR "health related quality of life" OR "Health Status" OR "health status" OR "HRQL" OR "patient outcomes" OR "PRO" OR "psychological" OR "psychosocial" OR "sexual functioning" OR "Sexuality" OR "Sexual behavior" OR "Social Interaction" OR "social functioning" OR "social wellbeing" OR "social" OR "Symptom Assessment" OR "symptom assessment" OR "symptom burden" OR "symptom distress" OR "Fatigue" OR "fatigue" OR "Pain" OR "pain"):ti,ab,kw) OR (("Metastatic Hormone sensitive Prostate Cancer" OR "mHSPC" OR "metastatic castration naive prostate cancer" OR (("Hormone sensitive" OR "Hormone naive" OR "hormone dependent" OR "castration sensitive" OR "castration naive") AND ("Prostatic Cancer" OR "prostate cancer" OR "prostate cancers" OR "prostate carcinoma" OR "prostate carcinomas" OR "prostate adenocarcinoma" OR "prostate adenocarcinomas" OR "prostate neoplasm" OR "prostate neoplasms" OR "prostate tumor" OR "prostate tumors" OR "prostate tumour" OR "prostate tumours" OR "prostate malignancy" OR "prostate malignancies" OR "prostatic cancer" OR "prostatic cancers" OR "prostatic carcinoma" OR "prostatic carcinomas" OR "prostatic adenocarcinoma" OR "prostatic adenocarcinomas" OR "prostatic neoplasm" OR "prostatic neoplasms" OR "prostatic tumor" OR "prostatic tumors" OR "prostatic tumour" OR "prostatic tumours" OR "prostatic malignancy" OR "prostatic malignancies") AND ("metastatic" OR "oligometastatic"))):ti,ab,kw AND ("Quality of Life" OR "Quality of Life" OR "Life Quality" OR "HR QoL" OR "HRQoL" OR "QoL" OR "Patient Reported Outcome" OR "Patient Reported Outcome" OR "Patient Reported Outcomes" OR "Patient Reported" OR "Anxiety" OR "anxiety" OR "Depression" OR "depression" OR "Distress Syndrome" OR "distress" OR "emotional" OR "Functional Status" OR "functional status" OR "health outcomes" OR "health related quality of life" OR "Health Status" OR "health status" OR "HRQL" OR "patient outcomes" OR "PRO" OR "psychological" OR "psychosocial" OR "sexual functioning" OR "Sexuality" OR "Sexual behavior" OR "Social Interaction" OR "social functioning" OR "social wellbeing" OR "social" OR "Symptom Assessment" OR "symptom assessment" OR "symptom burden" OR "symptom distress" OR "Fatigue" OR "fatigue" OR "Pain" OR "pain"):ti)) AND ("phase 3 clinical trial" OR "phase iii" OR "phase three" OR "phase 3" OR "phaseiii" OR "phasethree" OR "phase3"):ti,ab,kw  Manual exclusion of meeting abstracts |
| **Google Scholar** | Four queries  Manual exclusion of meeting abstracts  "Quality of Life"\|"Qol" "Metastatic" "Prostate Cancer" "androgen deprivation therapy"\|"ADT" "phase 3"\|"phase iii" -"non metastatic" -"nonmetastatic"  "PROs"\|"Patient Reported Outcomes" "Metastatic" "Prostate Cancer" "androgen deprivation therapy"\|"ADT" "phase 3"\|"phase iii" -"non metastatic" -"nonmetastatic"  "Quality of Life"\|"Qol" "mHPSC" "androgen deprivation therapy"\|"ADT" "phase 3"\|"phase iii" -"non metastatic" -"nonmetastatic"  "PROs"\|"Patient Reported Outcomes" "mHPSC" "androgen deprivation therapy"\|"ADT" "phase 3"\|"phase iii" -"non metastatic" -"nonmetastatic" |

**Legend Supplementary Table 2.**

Literature Search Strategy and Data Extraction.

A comprehensive/detailed search strategy was conducted and the following databases were searched: PubMed, Embase (OVID-version), Web of Science, Cochrane Library, and Google Scholar. Data extraction followed three subsequent steps of revisions following the Preferred Reporting Items for Systematic Review and Meta-Analyses (PRISMA) statement.

**Supplementary Table 3.** **Predefined clinical meaningful outcomes of PRO instruments used in RCTs**.

| **Outcome measurement** | **Questionnaire** | **Predefined clinical meaningful outcomes** |
| --- | --- | --- |
| **PATCH** | | |
| No predefined clinical meaningful outcomes. | | |
| **LATITUDE** | | |
| Clinical meaningful change | FACT-P Total Scales, PCS, TOI | Change of 6-10 points. Cited ref. **^61^** Cella et al. Estimating clinically meaningful changes for the Functional Assessment of Cancer Therapy – Prostate: results from a clinical trial of patients with metastatic hormone-refractory prostate cancer. Value Health 12: 124-129, 2009. |
| Time to pain progression | BPI-SF worst pain (item 3) | From randomization to the first date a patient experiences an increase by >30% from baseline in the BPI-SF worst pain intensity item (item 3) observed at 2 consecutive evaluations >4 weeks apart. |
| Time to pain interference | BPI-SF interference (scale) | Change of one half standard deviation of baseline BPI-SF interference score  Time to pain interference was defined as the time interval from randomization to the first date a patient experiences an increase of the threshold value specified by one half the sd of baseline scores from baseline in the BPI-SF pain interference item. |
| Time to fatigue intensity progression | BFI worst fatigue (item 3) | Time interval from randomization to the first date a patient experiences an increase by >2 points from baseline in the worst BFI intensity item (30 observed at 2 consecutive evaluations >4 weeks apart. |
| Fatigue interference progression | BFI-interference | An increase of >1.25 points from baseline in the average BFI interference score observed at 2 consecutive evaluations >4 weeks apart.  Cited ref: C.N. **^62^** Effect of abiraterone acetate on fatigue in patients with metastatic castration-resistant prostate cancer after docetaxel chemotherapy. Annals of Oncology 24(4): 1017-2025, 2013 |
| **CHAARTED** | | |
| Clinical meaningful change | FACT-P | Change of 6-10 points. Cited ref: **^61^** Cella et al. Estimating clinically meaningful changes for the Functional Assessment of Cancer Therapy – Prostate: results from a clinical trial of patients with metastatic hormone-refractory prostate cancer. Value Health 12: 124-129, 2009. |
| Clinical meaningful change | FACT-Taxane | Clinically meaningful difference in FACT-Taxane was a change of >1 standard error of measurement; Cited Ref: **^63^** Cella et al. Measuring the side effects of taxane therapy in oncology: the functional assessment of cancer therapy-taxane (FACT-taxane). Cancer 98:822-831, 2003 |
| Minimal important difference | FACIT-F | Minimal important difference (MID) was considered a change of > 3 points. Cited ref: **^64^** Nordin et al. Minimal important differences for fatigue patient reported outcome measures – a systematic review. BMC Med Res Methodol 16:62, 2016 |
| Minimal important difference | BPI-SF - Pain intensity | MID was a change of >2 points; Cited ref: Wong et al. **^65^**; **^66^** Mathias et al. Estimating minimally important differences for the worst pain rating of the Brief Pain Inventory-Short Form. J Support Oncol 9:72-78, 2011 |
| Minimal important difference | BPI-SF - Pain interference | MID >0.5 of the standard deviation; Cited ref: Wong et al, 2013, see above; Mathias et al. 2011, see above |
| **TITAN** | | |
| Minimal important difference | FACT-P Total Scales, PCS, TOI | Change of 6-10 points.^61^ Cited ref: Cella et al. Estimating clinically meaningful changes for the Functional Assessment of Cancer Therapy – Prostate: results from a clinical trial of patients with metastatic hormone-refractory prostate cancer. Value Health 12: 124-129, 2009. |
| Time to pain progression | BPI-SF worst pain (item 3) | From randomization to the first date a patient experiences an increase by >30% from baseline in the BPI-SF worst pain intensity item (item 3) observed at 2 consecutive evaluations >4 weeks apart. |
| Time to pain interference | BPI-SF interference (scale) | Change of one half standard deviation of baseline BPI-SF interference score.  Time to pain interference was defined as the time interval from randomization to the first date a patient experiences an increase of the threshold value specified by one half the sd of baseline scores from baseline in the BPI-SF pain interference item. |
| Time to fatigue intensity progression | BFI worst fatigue (item 3) | Time interval from randomization to the first date a patient experiences an increase by >2 points from baseline in the worst BFI intensity item (30 observed at 2 consecutive evaluations >4 weeks apart. |
| Fatigue interference progression | BFI-interference | An increase of >1.25 points from baseline in the average BFI interference score observed at 2 consecutive evaluations >4 weeks apart; Cited ref: C.N. ^62^ Sternberg et al. Effect of abiraterone acetate on fatigue in patients with metastatic castration-resistant prostate cancer after docetaxel chemotherapy. Annals of Oncology 24(4): 1017-2025, 2013 |
| **ARCHES** | | |
| Time to first clinically meaningful deterioration | FACT-P, EQ-5D-5L, BPI-SF, EORTC-QLQ-PR25, | The threshold for minimum clinically meaningful deterioration in score from baseline was 3 points for physical wellbeing, functional wellbeing, emotional wellbeing, family/social wellbeing, PCS, and FACT Advanced Prostate Symptom Index; 2 points for PCS pain-related score; 9 points for trial outcome index; 7 points for FACT-General; and 10 points for FACT-P. Pain progression was defined as a ≥2-point increase in BPI-SF pain score from baseline (except for pain interference, ≥1).  Cited refs:  BPI: **^67^** Cleeland & Ryan. Pain assessment: global use f the Brief Pain Inventory. Ann Acad Med Singapore 50: 920-8, 1997.  FACIT: Yost & Eton. Combining distribution- and anchor-based approaches to determine minimally important differences: the FACIT experience. Eval Health Prof 28: 172-91, 2005  EQ-5D**: ^68^** Pickard et al. Estimation of minimally important differences in EQ-5D utility and VAS scores in cancer. Health Qual Life Outcomes 5:70, 2007.  FACT-P: **^61^** Cella et al. Estimating clinically meaningful changes for the Functional Assessment of Cancer Therapy – Prostate: results from a clinical trial of patients with metastatic hormone-refractory prostate cancer. Value Health 12: 124-129, 2009.  Farrar et al. **^69^** Defining the clinically important difference in pain outcome measures. Pain 88: 287-94, 2000.  **^70^** Dworkin et al. Interpreting the clinical importance of treatment outcomes in chronic pain clinical trials: IMMPACT recommendations. J Pain 9:105-21, 2008. |
| **HORRAD** |  |  |
| Clinical meaningful difference | EORTC-QLQC30, EORTC-QLQ-PR25 | Change of > 10 points compared with baseline. No ref. |
| **STAMPEDE** |  |  |
| Clinical meaningful difference | EORTC -QLQC30 | Change of >4 points for global QoL. The size of the difference depends on the domain of the questionnaire, with a range of 3-6 points. Cited Ref: **^71^** Cocks K, King MT, Velikova G, et al: Evidence-based guidelines for determination of sample size and interpretation of the European Organisation for the Research and Treatment of Cancer Quality of Life Questionnaire Core 30. J Clin Oncol 29:89-96, 2011 |
| **ENZAMET** |  |  |
| Time to clinical deterioration | EORTC-QLQC30 | The time until a deterioration of >10 points in HQoL compared to baseline, clinical progression, treatment discontinuation or death from any cause.  Cited Ref**: ^71^**Cocks K, King MT, Velikova G, et al: Evidence-based guidelines for interpreting scores for the European organization for the Research and treatment of cancer quality of life questionnaire core 30. Eur J Cancer 48: 1713-1721, 2012. |
| **STAMPEDE Arm H** |  |  |
| Minimally important difference | EORTC -QLQC30 | To determine if any difference in average Global QoL observed between the treatment groups in the first two years on trial (using data from survivors at each time point) is clinically significant we will use the following categorisations, primarily informed by a 2011 paper by Cocks et al (J Clin Oncol 29:89-96) and a 1998 paper by Osabo et al (J Clin Oncol 16:139-144). If the 95% confidence interval around the point estimate for the difference in weighted averages does not include values in the range 0-4 we will conclude that there is evidence of a clinically significant difference. |

**Legend Supplementary Table 3. Predefined clinical meaningful outcomes of PRO instruments used in RCTs**. Clinical meaningful outcomes, or minimal important difference, clinically important difference, minimum clinically important difference, clinically significant difference, of PRO instruments used in the various RCTs.

**References**

61. Cella D, Nichol MB, Eton D, Nelson JB, Mulani P. Estimating clinically meaningful changes for the Functional Assessment of Cancer Therapy--Prostate: results from a clinical trial of patients with metastatic hormone-refractory prostate cancer. *Value Health* 2009; **12**(1): 124-9.

62. Sternberg CN, Molina A, North S, et al. Effect of abiraterone acetate on fatigue in patients with metastatic castration-resistant prostate cancer after docetaxel chemotherapy. *Ann Oncol* 2013; **24**(4): 1017-25.

63. Cella D, Peterman A, Hudgens S, Webster K, Socinski MA. Measuring the side effects of taxane therapy in oncology: the functional assesment of cancer therapy-taxane (FACT-taxane). *Cancer* 2003; **98**(4): 822-31.

64. Nordin Å, Taft C, Lundgren-Nilsson Å, Dencker A. Minimal important differences for fatigue patient reported outcome measures-a systematic review. *BMC Med Res Methodol* 2016; **16**: 62.

65. Wong K, Zeng L, Zhang L, et al. Minimal clinically important differences in the brief pain inventory in patients with bone metastases. *Support Care Cancer* 2013; **21**(7): 1893-9.

66. Mathias SD, Crosby RD, Qian Y, Jiang Q, Dansey R, Chung K. Estimating minimally important differences for the worst pain rating of the Brief Pain Inventory-Short Form. *J Support Oncol* 2011; **9**(2): 72-8.

67. Cleeland CS, Ryan KM. Pain assessment: global use of the Brief Pain Inventory. *Ann Acad Med Singap* 1994; **23**(2): 129-38.

68. Pickard AS, Neary MP, Cella D. Estimation of minimally important differences in EQ-5D utility and VAS scores in cancer. *Health Qual Life Outcomes* 2007; **5**: 70.

69. Farrar JT, Portenoy RK, Berlin JA, Kinman JL, Strom BL. Defining the clinically important difference in pain outcome measures. *Pain* 2000; **88**(3): 287-94.

70. Dworkin RH, Turk DC, Wyrwich KW, et al. Interpreting the clinical importance of treatment outcomes in chronic pain clinical trials: IMMPACT recommendations. *J Pain* 2008; **9**(2): 105-21.

71. Cocks K, King MT, Velikova G, Martyn St-James M, Fayers PM, Brown JM. Evidence-based guidelines for determination of sample size and interpretation of the European Organisation for the Research and Treatment of Cancer Quality of Life Questionnaire Core 30. *J Clin Oncol* 2011; **29**(1): 89-96.

**Supplementary Table 4. Level of patient-reported outcomes (PRO) reporting according to the ISOQOL recommended standards from a total of eight phase III randomized controlled trials**

| **ISOQOL recommended standards** | |
| --- | --- |
| **PRO Reporting items** | **Total (n=8)**  **n (%)** |
| The PRO should be identified in the abstract as a primary or secondary outcome. | 8 (100) |
| The PRO hypothesis should be stated and relevant domains identified, if applicable | 2 (25) |
| The mode of administration, including the person completing the PRO and methods of data collection (paper, telephone, electronic, other) should be described. | 7 (87.5) |
| The rationale for choice of the PRO instrument used should be provided. | 5 (62.5) |
| Evidence of PRO instrument validity and reliability should be provided or cited, if available. | 8 (100) |
| The intended PRO data collection schedule should be provided. | 8 (100) |
| PROs should be identified in the trial protocol; post-hoc analyses should be identified. | 8 (100) |
| The status of PRO as either a primary or secondary outcome should be stated. | 8 (100) |
| There should be evidence of appropriate statistical analysis and tests of statistical significance for each PRO hypothesis tested*. | 2 (100%) |
| The extent of missing data should be stated†. | 8 (100) |
| Statistical approaches for dealing with missing data should be explicitly stated†. | 7 (87.5) |
| The reasons for missing data should be explained. | 4 (50) |
| A flow diagram or a description of the allocation of participants and those lost to follow-up should be provided for PROs specifically. | 8 (100) |
| The study patients’ characteristics should be described, including baseline PRO scores. | 8 (100) |
| The limitations of the PRO components of the trial should be explicitly discussed. | 7 (87.5) |
| Generalizability issues uniquely related to the PRO results should be discussed, if applicable. | 5 (62.5) |
| The clinical significance of the PRO findings should be discussed. | 8 (100) |
| The PRO results should be discussed in the context of the other clinical trial outcomes | 8 (100) |

**Legend Supplementary Table 4. Level of patient-reported outcomes (PRO) reporting according to the ISOQOL recommended standards from a total of eight phase III randomized controlled trials.**

^*^ Only where applicable.

^†^These items were originally combined in the ISOQOL recommended standards but have been split for the purpose of our work.

**Supplementary Table 5. Geographical distribution of mHSPC RCTs with HRQoL publications (Jan 2015 - Sept 2024).**

| **RCT** | **Geographical distribution** | **Nr. of countries or sites** | **Time period; nr of pts** |
| --- | --- | --- | --- |
| **Pharma sponsored Studies** | | | |
| **LATTITUDE**  (Janssen R&D) | North and Latin America, Europe, and the Asia–Pacific region | 235 sites in 34 countries in Europe, the Asia–Pacific region, Latin America, and Canada. | 12 Feb2013-11Dec 2014;  1,119 pts |
| **TITAN**  (Janssen R&D) | North and South America, Europe, Asia and Australia, | 260 sites in 23 countries. | 15 Dec 2015-25 July 2017;  1,052 pts |
| **ARCHES**  (Astellas Pharma Inc. and Pﬁzer Inc.) | North and Latin America, Europe, and Asia | 202 centers, in 24 countries | 21 Mar 2016-12 Jan 2018; 1,150 pts |
| **Academic/Investigator-Initiated Studies** | | | |
| **CHAARTED**  (ECOG) | USA | 1 country (USA) | July 2006-Sept 2012;  790 pts |
| **PATCH**  (MRC) | United Kingdom (UK) | 1 country | Aug 2007-Oct 2015;  727 pts |
| **HORRAD**  (Academia) | The Netherlands | 1 country, 28 sites | Nov 2004 -Sept 2014;  446 pts |
| **STAMPEDE**  Abiraterone  (MRC) | United Kingdom (UK) and Switzerland | 2 countries (111 U.K. and 5 Swiss sites) | 15 Nov 2011-17 Jan 2014;  1,917 pts |
| **STAMPEDE**  Docetaxel  (MRC) | United Kingdom (UK) and Switzerland | 2 countries; more than 100 U.K. and Swiss sites | 5 Oct 2005- 31 Mar 2013;  2,962 pts. |
| **STAMPEDE EBRT**  (MRC) | United Kingdom (UK) and Switzerland | 2 countries, 117 sites in the UK and Switzerland. | 22 Jan 2013-2 Sept 2016;  2,061 pts |
| **ENZAMET**  (ANZUP) | North America, Ireland, United Kingdom (UK), Australia and New Zealand | 83 sites | March 2014-March 2017;  1,125 pts |

**Legend Supplementary Table 5.** Geographical distribution, number of countries or sites, and time period in which the study was executed plus total number of patients in the study are shown.

Abbreviations: MRC=Medical Research Council. EBRT= External Beam Radiation Therapy. ANZUP= The Australian and New Zealand Urogenital and Prostate Cancer Trials Group. Pts Patients.

**Supplementary Table 6. RCTs of intensification of androgen deprivation therapy with ARPIs or chemotherapy (doublets and triplets) in mHSPC showing improved**

**survival (2015-2024)**

| **Study name/**  **first author** | **1st year of publication** | **EC** | **CG** | **No. of pts** | **Synchronous M1, %** | **High-volume,**  **%** | **Prior ADT** | **Anti-androgens**  **with ADT** | **Prior/concurrent docetaxel** | **Outcomes** | **Median rPFS**  **ARPI +ADT doublets** | **HR for OS (95%CI)** | **Median**  **FU mo** |
| --- | --- | --- | --- | --- | --- | --- | --- | --- | --- | --- | --- | --- | --- |
| **CHAARTED**  ^28^ ^72^ | 2015 | DOC + ADT | ADT | 790 | 73% | 65% | Up to 3.9 mo (median 1mo) | Both arms | Exclusion | OS, PFS |  | 0.72 (0.59–0.89) | 53.7 |
| **LATITUDE**  ^27^ ^73^ | 2017 | AAP + ADT | ADT+ dual placebos | 1,199 | 100% | 100% High risk | Up to 3 mo (median 1mo) | 62% | Exclusion | OS, PFS | 33.0 *vs* 14.8 months | 0.66 (0.56–0.78) | 51.8 |
| **STAMPEDE**  ^33^ ^74^ | 2017 | AAP + ADT | ADT | 990 | 49% | (41% M0) | Up to 3 mo | 94% | Exclusion | OS, PFS |  | 0.61 (0.49–0.75) | 40 |
| **STAMPEDE**  ^32^ ^75^ | 2016 | DOC + ADT | ADT | 1,086 | 49% | (41% M0) | Up to 3 mo | 94% | Exclusion | OS, PFS |  | 0.81 (0.69–0.95) | 78.2 |
| **TITAN**  ^29^ ^76^ | 2019 | APA + ADT | ADT+ placebo | 1,052 | 81% | 63% | Up to 6 mo | Experimental. arm only | Prior docetaxel up to 6 cycles 11% | OS, PFS | NR *vs* 22.1 months | 0.65 (0.53–0.79) | 44 |
| **ARCHES**  ^30^ ^37^ | 2019 | ENZ + ADT | ADT+ placebo | 1,150 | 67% | 63% | Up to 6 mo (70% ⩽3mo) | Experimental. arm only | Prior docetaxel up to 6 cycles 17.9% | OS, PFS | NR *vs* 19.0 months | 0.66 (0.53–0.81) | 44.6 |
| **ENZAMET**  ^36^ ^77^ | 2019 | ENZ + DOC + ADT | DOC + ADT | 503 | 67% | 53% | Up to 3 mo | Both arms | Prior docetaxel up to 2 cycles in 15.8%; concurrent in 44.7% | OS, PFS |  | 0.7 (0.58–0.84) | 68 |
|  |  | ENZ + ADT | ADT | 622 |  |  |  |  |  |  |  |  |  |
| **ARASENS**  ^44^ | 2022 | DAR + DOC + ADT | DOC + ADT | 1306 | 86% | NE | Up to 12 wks | Experimental. arm only | Exclusion | OS, PFS |  | 0.68 (0.57–0.80) | ≈43.7 |
| **PEACE-1**  ^45^ | 2022 | AAP + DOC + ADT | DOC + ADT | 710 | 100% | 64% | Up to 3 mo | No | Concurrent 60% | OS, PFS |  | 0.75 (0.55–0.95) | 45.7 |

**Legend Supplementary Table 6**. Intensification of androgen deprivation therapy (ADT) with androgen receptor pathway inhibitor (ARPI) agents (abiraterone [AA], apalutamide [APA], and enzalutamide [ENZ] or chemotherapy (docetaxel [DOC]) for patients with metastatic hormone-sensitive prostate cancer (mHSPC) which have demonstrated delay disease progression and prolonged patient survival. RCT Randomized controlled trial, EC=experimental group. CG=control group. Pts Patients, Wks Weeks, Mo months, Synchronous M1=patients who presented with synchronous metastatic disease (“de novo metastatic disease”), High Volume (HV) according to CHAARTED high volume criteria: >4 bone metastases (at least one outside the spine or pelvis AND/OR Visceral metastases. High Risk according to LATITUDE high risk criteria, i.e. two or more of the following criteria: >3 bone metastases, Gleason score >8, Visceral metastases (not including lymph node metastases). rPFS radiographic Progression Free Survival; HR Hazard ratio OR: HR = hazard ratio for risk of death and 95% CI; CI = confidence interval; OS Overall survival, CI Confidence interval, NE, not evaluable, DOC Docetaxel, ADT Androgen deprivation therapy, AAP Abiraterone acetate (AA) and P, prednisone, APA Apalutamide, DAR Darolutamide, ENZ Enzalutamide, mHSPC metastatic hormone-sensitive prostate cancer. Patients treated with abiraterone in LATITUDE received prednisone 5 mg daily and in STAMPEDE received prednisolone 5 mg orally once per day, Patients treated with docetaxel in STAMPEDE received 10 mg of prednisolone/prednisone daily plus prednisolone/prednisone as premedication during the 6 cycles of docetaxel chemotherapy.

Doublets (combination of ADT plus another drug) are highlighted in green, triplets (combinations of ADT plus 2 other drugs, vs doublet of ADT plus docetaxel) are highlighted in pink.

**References**

72. Kyriakopoulos CE, Chen YH, Carducci MA, et al. Chemohormonal Therapy in Metastatic Hormone-Sensitive Prostate Cancer: Long-Term Survival Analysis of the Randomized Phase III E3805 CHAARTED Trial. *J Clin Oncol* 2018; **36**(11): 1080-7.

73. Fizazi K, Tran N, Fein L, et al. Abiraterone acetate plus prednisone in patients with newly diagnosed high-risk metastatic castration-sensitive prostate cancer (LATITUDE): final overall survival analysis of a randomised, double-blind, phase 3 trial. *Lancet Oncol* 2019; 20(5): 686-700.

74. Hoyle AP, Ali A, James ND, et al. Abiraterone in "High-" and "Low-risk" Metastatic Hormone-sensitive Prostate Cancer. *Eur Urol* 2019; **76**(6): 719-28.

75. Clarke NW, Ali A, Ingleby FC, et al. Addition of docetaxel to hormonal therapy in low- and high-burden metastatic hormone sensitive prostate cancer: long-term survival results from the STAMPEDE trial. *Ann Oncol* 2019; **30**(12): 1992-2003.

76. Chi KN, Chowdhury S, Bjartell A, et al. Apalutamide in Patients With Metastatic Castration-Sensitive Prostate Cancer: Final Survival Analysis of the Randomized, Double-Blind, Phase III TITAN Study. *J Clin Oncol* 2021; **39**(20): 2294-303.

77. Sweeney CJ, Martin AJ, Stockler MR, et al. Testosterone suppression plus enzalutamide versus testosterone suppression plus standard antiandrogen therapy for metastatic hormone-sensitive prostate cancer (ENZAMET): an international, open-label, randomised, phase 3 trial. *Lancet Oncol* 2023; 24(4):323-334.

**SUPPLEMENTARY APPENDIX 1**

**Protocol for Systematic Review of the Effects of New Systemic Treatments on Quality of Life in phase III randomized controlled trials in patients diagnosed with metastatic hormone sensitive prostate cancer (mHSPC).**

Available in the PROSPERO database (CRD42023470698).

**Research Question**

To determine the effect of upfront Systemic Treatments compared to that of standard androgen deprivation therapy (ADT) on the Quality of life (QoL) in patients diagnosed with metastatic hormone sensitive prostate cancer (mHSPC).

**Objective**

To systematically review health-related quality of life (HRQoL, QoL) publications since 2015 of phase III randomized controlled clinical trials (RCTs) in mHSPC with the aim to cross- compare QoL results and assess their usefulness to support clinical decision-making.

**Authors:**

Anne-Laurien Bout, Department of Urology, Leiden University Medical Center.

Cristina Alvarez Gomez de Segura, Department of Oncology, Leiden University Medical Center.

Peter-Paul M. Willemse, Department of Urology, Cancer Center, University Medical Center Utrecht, Utrecht, The Netherlands.

Jan Schoones, Directorate Research Policy, Leiden University Medical Center.

Adam Cohen, Center of Human Drug Research (CHDR), Leiden, and Clinical Pharmacology, Leiden University Medical Center, Leiden, The Netherlands.

Sahar Barjesteh van Waalwijk van Doorn-Khosrovani, CZ Health insurance, Tilburg.

Fabio Efficace, Italian Group for Adult Hematologic Diseases (GIMEMA) Data Center and Health Outcomes Research Unit, Rome, Italy.

Susanne Osanto, Department of Clinical Oncology, Leiden University Medical Center.

**Protocol Version Number:1**

Date: 2023.09.27

**Introduction**

Health-related quality of life data (HRQoL, QoL) obtained by validated tool questionnaires measure the value of cancer treatment through the lens of the patient. HRQoL is a dynamic endpoint of treatment benefit rather than overall survival (OS) endpoint and could potentially support the benefit/risk ratio of intervention treatment evaluation. To this end HRQoL should be a robust method, preferably with measurable elements, next to clinical outcomes such as progression-free and overall survival (PFS, OS). Key questions are whether the investigational treatment arm improves QoL or delays deterioration and whether QoL is better, worse than, or non-inferior to QoL associated with the standard of care (SOC).

**Clinical relevance of the systematic review**

For decades, androgen deprivation therapy (ADT) was the mainstay in **metastatic hormone-sensitive prostate cancer (mHSPC).** Since 2015 a number of phase III RCTs demonstrated OS benefit of a number of investigational combinations to ADT compared to ADT ± placebo, showing similar hazard ratios of death. This changed the treatment landscape of mHSPC but lack of head-to-head comparisons between investigational treatments in the presence of greatly varying drug costs posed challenges to clinicians in clinical decision-making. Availability of statistically and, more importantly, clinically significant, differences in QoL outcomes could help in prioritizing between multiple treatment options with similar toxicity and OS benefit.

**Aims and objectives**

The aim is to perform a systematic review of all RCT’s reporting Quality of life of patients treated for metastatic hormone sensitive prostate cancer (mHSPC).

The objectives are:

1. To investigate HRQoL methodology of mHSPC RCTs using The International Society for Quality of Life Research (ISOQOL recommendations.
2. To compare statistical differences in QoL outcomes between treatment arms within individual RCTs and across RCTs
3. To assess whether QoL results are useful to support clinical decision making in mHSPC.

**Trial Eligibility Criteria**

Types of studies

Phase III RCT’s will be included.

All prospective case cohort studies, non-randomised comparative studies and retrospective studies will be excluded.

*Types of participants*

1. Adult men >18 years of age with either synchronous or metachronous metastatic hormone sensitive prostate cancer who are undergoing systemic treatment.

*Inclusion Criteria*

1. Health-related quality of life (HRQoL, QoL) publications of phase III RCT’s investigating new systemic treatment in comparison to androgen deprivation therapy (ADT) standard of care in mHSPC.
2. Relevant QoL publications between 2015 and 2023.

*Exclusion Criteria*

1. Absence of reporting QoL assessment tools.
2. Absence of specific upfront Systemic Treatment information.

*Types of outcomes measures*

The primary outcome is:

Effects of new systemic treatment on Quality of Live (QoL) in mHSPC and to compare these effects on QoL between the different treatment arms.

The secondary outcomes are:

1. Evaluation of the QoL tools used.
2. Evaluation of the methodology and quality of HRQoL in mHSPC phase III RCTs according to The International Society for Quality of Life Research (ISOQOL) recommendations.
3. Comparison of QoL outcomes between the different QoL assessment tools in the investigational treatment arm versus the standard treatment arm of the phase III RCT’s on new systemic agents in mHSPC.
4. To assess whether QoL results from these phase III RCTs are useful to support clinical decision making in mHSPC.

**Material and Methods**

*Literature Search*

A comprehensive/detailed search strategy will be conducted and the following databases will be searched: PubMed, Embase (OVID-version), Web of Science, Cochrane Library, and Google Scholar for all relevant publications published in English between 2015 and 2023.

Data extraction follows three subsequent steps of revisions according to the Preferred Reporting Items for Systematic Review and Meta-Analyses (PRISMA) statement^1^.

Meeting abstracts will not be included.

The literature search will be carried out based on the search strategy provided in Appendix 1.

***Data collection and analysis***

*Selection of studies*

Following de-duplication, two review authors will independently screen the titles and abstracts of identified records for eligibility. The full text of all potentially eligible records will be retrieved and screened independently by two review authors using a standardised form, linking together multiple records of the same study in the process. Any disagreements will be resolved by discussion or by consulting a third review author. The study selection process will be described using a PRISMA flow diagram^1^

*Data extraction and management*

Two review authors will independently extract outcome data. Study characteristics will be extracted by one review author and a second review author will check data extractions for accuracy. Any disagreements will be resolved by discussion or by consulting a third review author. A standardised data extraction form will be developed and piloted before its use. In case of any incompletely reported data, study authors will be contacted.

*Assessment of risk of bias in included studies*

The risk of bias of each study will be assessed by two review authors working independently. Any disagreements will be resolved by consensus or by consulting a third review author if necessary. The quality of included publications will be assessed using the risk of bias tool of the Revised Cochrane risk-of-bias tool for randomized trials (RoB 2)^2^

*Dealing with missing data*

We will include studies that investigate the QoL in patients with mHSPC treated with new systemic treatment in comparison to the then standard of care, i.e. androgen deprivation therapy (ADT). We will not impute missing data. In case of any incompletely reported data, we will attempt to contact the study authors.

*Assessment of publication bias*

The review authors will aim to minimise potential biases by conducting a comprehensive literature search for eligible studies. Publication bias will be examined for each RCT published.

*Data synthesis*

The methodological QoL assessments was reviewed by using The International Society for Quality of Life Research (ISOQOL) checklist of 18 items^3, 4^  for evaluating HRQoL outcomes in cancer clinical trials. Investigators assessed QoL instruments used and whether QoLs were part of the primary aims of the trials. Quality of data, missing data reported, and analyses performed were reviewed.

For cross-study comparisons, baseline QoL of the patient populations across RCTs was studied to assess QoL homogeneity of the different mHSPC trial populations.

**Acknowledgements**

Acknowledgements will be stated in agreement with the rules set by the peer review journal that will publish this systematic review.

**Contributions of authors**

Contributions of authors will be stated in agreement with the rules set by the peer review journal that will publish this systematic review.

**Declaration of conflicts of interest**

Declaration of conflicts of interest will be stated in agreement with the rules set by the peer review journal that will publish this systematic review.

**Administrative Aspects**

Literature Search

A comprehensive/detailed search strategy will be conducted by a trained medical librarian (JWS) together with two reviewers. The databases searched are PubMed, Embase (OVID-version), Web of Science, Cochrane Library, and Google Scholar. The query consisted of the combination of the following four concepts: 1) Metastatic Hormone Sensitive Prostate Cancer, 2) Androgen Deprivation Therapy, 3) Quality of Life or Patient Reported Outcome Measures and 4) Phase 3 Trials.

Relevant keyword variations in the controlled vocabularies of the various databases, and the free text word variations of these concepts. Results will be limited to articles published from 2015 till 2023, in the English language, while meeting abstracts will be excluded. Data extraction follows three subsequent steps of revisions following the Preferred Reporting Items for Systematic Review and Meta-Analyses (PRISMA) statement^1^ (Eligibility screening of publications will be conducted independently by two reviewers to identify the final included PRO publications of RCTs. In the first step, titles and abstracts of search results based on inclusion and exclusion criteria will be screened. References will be retrieved in full text and evaluated. Disagreements will be resolved by consensus, discrepancies reconciled by discussion or a third review.

Data Quality Control Committee

Data quality control will be assured by Susanne Osanto

Publication of the Results and Authorship

Publication of results will be in a peer review journal and authorship based on ICMJE Authorship Rules.

Timelines

Literature search and abstract and full text screening will be conducted and data extraction and analysis performed. A preliminary search to test the selection process has been performed. Formal screening of search results against eligibility criteria, data extraction, analysis of the data, risk of bias (quality) assessment and drafting of the systematic review article will be performed thereafter.

Data analysis

This systematic review will be conducted through altruistic donation of time and knowledge from involved parties.

References

1. Page, M. J., McKenzie, J. E., Bossuyt, P. M., Boutron, I., Hoffmann, T. C., Mulrow, C. D., Shamseer, L., Tetzlaff, J. M., Akl, E. A., Brennan, S. E., Chou, R., Glanville, J., Grimshaw, J. M., Hróbjartsson, A., Lalu, M. M., Li, T., Loder, E. W., Mayo-Wilson, E., McDonald, S., . . . Moher, D. (2021). The PRISMA 2020 statement: an updated guideline for reporting systematic reviews. Bmj, 372, n71. https://doi.org/10.1136/bmj.n71
2. Sterne JAC, Savović J, Page MJ et al. RoB 2: a revised tool for assessing risk of bias in randomised trials. BMJ. 2019 Aug 28;366:l4898. doi: 10.1136/bmj.l4898. PMID: 31462531.
3. Brundage, M., Blazeby, J., Revicki, D., Bass, B., de Vet, H., Duffy, H., Efficace, F., King, M., Lam, C. L., Moher, D., Scott, J., Sloan, J., Snyder, C., Yount, S., & Calvert, M. (2013). Patient-reported outcomes in randomized clinical trials: development of ISOQOL reporting standards. Qual Life Res, 22(6), 1161-1175. <https://doi.org/10.1007/s11136-012-0252-1>
4. Efficace, F., Bottomley, A., Osoba, D., Gotay, C., Flechtner, H., D'Haese, S., & Zurlo, A. (2003). Beyond the development of health-related quality-of-life (HRQOL) measures: a checklist for evaluating HRQOL outcomes in cancer clinical trials--does HRQOL evaluation in prostate cancer research inform clinical decision making? J Clin Oncol, 21(18), 3502-3511. <https://doi.org/10.1200/jco.2003.12.121>

**SUPPLEMENTARY APPENDIX 2.**

**Clinical Study Information to Table 1. Summary of HRQoL studies published between Jan 2015 – Sept 2024 of phase III RCTs in mHSPC.**

PATCH trial: Only 40% had metastatic disease (mHSPC), while 60% had locally advanced disease (nmHSPC). Phase 2 and 3 part of the study were combined. tE2 was delivered via three or four transcutaneous patches containing oestradiol 100 g/24 h, versus LHRHa for ADT. LHRHa=LHRH antagonist, was administered as per local practice.

LATITUDE trial: High-risk was defined as meeting at least two of three criteria: (i) Gleason score ≥8, (ii) presence of ≥3 lesions on bone scan, or (iii) presence of measurable visceral lesions. ADT consisted of LHRH or surgical castration. After the results of the first interim analysis, the LATITUDE study was unblinded and patients in the placebo group were allowed to cross over to receive abiraterone acetate and prednisone plus ADT treatment in accordance with a protocol amendment (February 2017) in an open-label extension of the study.

CHAARTED trial: High-volume was defined as: Visceral Metastases AND/OR ≥4 Bone Lesions (with ≥1 beyond the vertebral bodies and pelvis). ADT (LHRH receptor agonist or an LHRH receptor antagonist or orchiectomy); antiandrogens were given at the investigators’ decision.

TITAN trial: After unblinding of the study, patients on ADT (with placebo) were allowed to cross over to APA.

ARCHES trial: Patients were stratified by disease volume (low v high per CHAARTED criteria) and prior docetaxel chemotherapy for prostate cancer. On the basis of the primary analysis results eligible patients were offered the opportunity to transition to an open-label extension.

HORRAD trial in mHSPC with the primary tumour in *situ.* Addition of RT to the primary (prostate RT) did not improve OS, but an unplanned subset analysis demonstrated that patients with low volume disease had a non-significant longer OS (HR 0.68; 95% CI, 0.42–1.10).

STAMPEDE trial: Only 59% of the patients had metastatic while 41% had non-metastatic disease. Docetaxel was given for six 3-weekly cycles with prednisolone (10 mg) daily, and standard premedication before each injection.

STAMPEDE trial Radiotherapy arm H: 41% of patients was non-metastatic and 59% was metastatic: Addition of RT to the primary (prostate RT) demonstrated a statistically significant benefit in overall survival (OS) for patients with a low metastatic burden.

ENZAMET trial: The control arm received ADT + a standard nonsteroidal antiandrogen drug (e.g. bicalutamide. Docetaxel was administered without prednisone or prednisolone; Up to two cycles of docetaxel were permitted before randomisation.
